# Understanding the Role of Hypothalamic–Pituitary–Gonadal Axis in Multiple Sclerosis and Experimental Autoimmune Encephalomyelitis

**DOI:** 10.3390/biomedicines14081796

**Published:** 2026-08-10

**Authors:** Ana Milošević, Marija M. Janjic, Dunja Dimitrijevic, Ivana Bjelobaba

**Affiliations:** Institute for Biological Research “Siniša Stanković”—National Institute of the Republic of Serbia, University of Belgrade, 11108 Belgrade, Serbia; marija.janjic@ibiss.bg.ac.rs (M.M.J.); dunja.dimitrijevic@ibiss.bg.ac.rs (D.D.); ivana.bjelobaba@ibiss.bg.ac.rs (I.B.)

**Keywords:** multiple sclerosis, experimental autoimmune encephalomyelitis, hypothalamic-pituitary-gonadal axis, sex hormones, testosterone, estradiol, progesterone

## Abstract

Multiple sclerosis (MS) is a chronic inflammatory disease of the central nervous system, mostly occurring during the reproductive period, and it is diagnosed more frequently in women. The sex biases in the incidence and course of both MS and its most commonly used animal model, experimental autoimmune encephalomyelitis (EAE), seem to be driven by fluctuations in sex hormones. The regulation of sex hormone synthesis and release relies on a dynamic hierarchical system—the hypothalamic–pituitary–gonadal (HPG) axis. This review provides an overview of the main characteristics of MS and EAE pathologies and explains the structure and function of the HPG axis in order to better understand the sex-specific features of MS and EAE. The particular role of sex hormones as culprits in the sex differences in these pathologies is covered, as well as the disturbances to the HPG axis that arise as a consequence of MS/EAE course. Finally, the use of sex hormones as potential adjuvant therapeutics for MS is discussed. Given the complexity and dynamism in the regulation of the HPG axis, an individual, holistic approach should be taken when considering sex hormones for the treatment of MS patients.

## 1. Introduction

### 1.1. Multiple Sclerosis

Multiple sclerosis (MS) is the most widespread chronic inflammatory disease of the central nervous system (CNS), affecting about two [1] to three [2] million people worldwide. It is usually diagnosed during the reproductive period, between 20 and 40 years of age. The main pathologic features of MS are the demyelinating lesions in different CNS regions, leading to a wide array of sensory and motor dysfunctions [3].

The etiology of MS is not completely understood, but most probably, the disease is a consequence of genetic predisposition, i.e., the polymorphisms in genes coding for molecules involved in immune reactions [4], triggered by environmental cues, such as the extent of sun exposure, smoking or obesity [5]. Epstein–Barr infection after puberty has been linked with increased risk for MS as well [6].

In clinical practice, several types of MS have been defined. Relapsing-remitting MS (RRMS) affects about 85% of MS patients, and it is characterized by symptom breakout episodes, when new inflammatory demyelinating lesions are formed/activated, after which periods of remission occur. Between 15 and 30% of RRMS patients will slowly develop secondary-progressive MS, characterized by a gradual progression of symptoms, without remission phases. During the symptoms’ progression, additional acute exacerbations can occasionally happen. Primary-progressive MS features a constant neurological decline since the appearance of the first symptoms, while acute inflammation episodes are absent. The most recently identified type of MS, the clinically isolated syndrome, is characterized by inflammation and demyelination but lacks dissemination in time [7]. However, the 2024 revisions of the McDonald criteria exclude dissemination in time as the necessary component for diagnosing MS [8]. Among the diverse symptoms of MS, the most common ones are the unilateral loss of vision, limb paresis, numbness, poor movement coordination, as well as fatigue, vertigo and neuropathic pain. Of note, the symptoms can also include dysfunction in the urinary and gastrointestinal tracts [9,10]. The symptoms of MS arise as a consequence of demyelinating lesions that occur in the white and gray matter of the CNS. Active demyelinating lesions are surrounded by inflammatory infiltrates consisting of T cells and macrophages or monocytes [11]. Aside from the predominant CD8^+^ T cells, CD4^+^ T cells are also included in the infiltrates, as well as B cells [12,13,14].

The pathogenesis of MS is supported by one of the following two models. The “outside-in” hypothesis encompasses inflammation as the pioneering event in the development of the disease: myelin-specific T lymphocytes are activated after encountering a pathogen, they then migrate through the blood–brain barrier, and become reactivated by resident antigen-presenting cells in the CNS. Demyelination and neuronal death are the consequences of these processes [15,16]. On the other hand, the “inside-out” hypothesis proposes myelin degradation as the triggering event of the peripheral inflammatory reaction toward myelin proteins, causing the activation of T and B cells, which then migrate to the CNS [17,18].

### 1.2. Experimental Autoimmune Encephalomyelitis

Experimental autoimmune encephalomyelitis (EAE) is the most widely used animal model of MS. It is usually induced in several strains of rats and mice, which are genetically susceptible to developing the disease, such as Dark Agouti and Lewis rat strains, and C57BL/6 and SJL mouse strains. EAE can be induced by active immunization—via an injection of myelin antigens, usually mixed with complete Freund’s adjuvant—or passively by an adoptive transfer of encephalitogenic T lymphocytes isolated from a previously immunized animal. EAE course varies depending on the species, strain and age of the animals, as well as on the method of induction. In mice and rats, the most common types are acute, relapsing–remitting and chronic EAE [19,20].

Similar to MS, EAE pathogenesis is characterized by disruption of the blood–brain barrier, the infiltration of immune cells into the CNS parenchyma and neuroinflammation, which leads to demyelination and progressive loss in motor functions. The main role in EAE immunopathogenesis is played by CD4^+^ T cells [11]. Namely, naïve CD4^+^ T cells are presented with myelin antigens in the lymph nodes, which causes their activation, more specifically their differentiation into effector phenotypes of T helper (Th) cells. Depending on the surrounding molecular milieu, i.e., the cytokines released by the antigen-presenting cells, naïve Th cells will differentiate into Th1, Th2 or Th17 phenotypes [21]. Th17 cells have a crucial role in the effector phase of EAE [11]. After passing the blood–brain barrier, Th cells are presented with myelin antigens by the resident antigen-presenting cells of the CNS—most commonly perivascular macrophages or microglia, but also astrocytes and dendritic cells [19]. This triggers the reactivation of T cells, leading to a cascade of events that result in glial cell activation, demyelination and axonal loss. Besides Th1 and Th17 cells, EAE pathogenesis includes other cells of adaptive immunity, albeit to a lesser extent—CD8^+^ lymphocytes, B cells, and regulatory T cells that are important for the resolution of neuroinflammation [12,22]. Besides immune cells, the crucial players in the effector phase of EAE and the CNS damage are astrocytes and microglia. During the effector phase of EAE, both astrocytes and microglia become activated, and depending on their activation profile, they acquire neuroinflammatory or neuroprotective phenotypes, which contribute to different phases of the disease development and resolution [23,24,25].

Despite many similarities between MS and EAE, one should consider some differences between these two pathologies, such as the involvement of different types of T lymphocytes: MS lesions are rich in CD8^+^ T cells, while in EAE, the main immune cells in lesions are CD4^+^ T lymphocytes [14]. Furthermore, demyelination is usually limited to the spinal cord in EAE, while in MS it also occurs in the cerebral and cerebellar cortex [18,26]. Also, recapitulating the progressive form of MS in animal models is still challenging [27].

However, it is important to highlight that EAE is the MS model that represents most of the pathophysiological aspects of MS [28], unlike any other model. This has led to the development of a great number of therapeutic approaches [12], keeping EAE an indispensable model in MS research.

It should be emphasized that EAE is not the only experimental model used in preclinical MS research. In particular, the investigation of the inside-out hypothesis relies heavily on toxin-induced demyelination models, which provide valuable insights into primary oligodendrocyte injury and myelin degeneration. Viral models like Theiler’s murine encephalomyelitis virus model are used to model some aspects of progressive MS, like chronic progressive demyelination. The use of genetic models with mutations in genes for myelin proteins or the use of humanized mice [29] has also increased in the past two decades. In addition to in vivo animal models, preclinical MS research also employs a wide range of in vitro and ex vivo approaches, enabling detailed mechanistic studies of disease processes and facilitating the evaluation of potential therapeutic strategies [30].

Among various physiological systems affected by neuroinflammatory processes occurring during both MS and EAE, the hypothalamic–pituitary–gonadal (HPG) axis has emerged as a critical yet underexplored player. Besides its primary role in the regulation of reproduction, the HPG axis exerts significant influence on immune modulation and neuroprotection. The disruption of HPG axis homeostasis in MS or EAE [31,32,33,34], as well as other neuroendocrine systems affected by these diseases, such as the hypothalamic–pituitary–adrenal (HPA) axis [35,36,37], could therefore impact disease susceptibility, progression, and symptomatology. Thus, understanding the interactions between the HPG axis and autoimmune neuroinflammation holds promise for advancing knowledge on MS pathophysiology and identifying novel therapeutic strategies.

### 1.3. Hypothalamic–Pituitary–Gonadal Axis: Structure and Function

**Figure 1:**
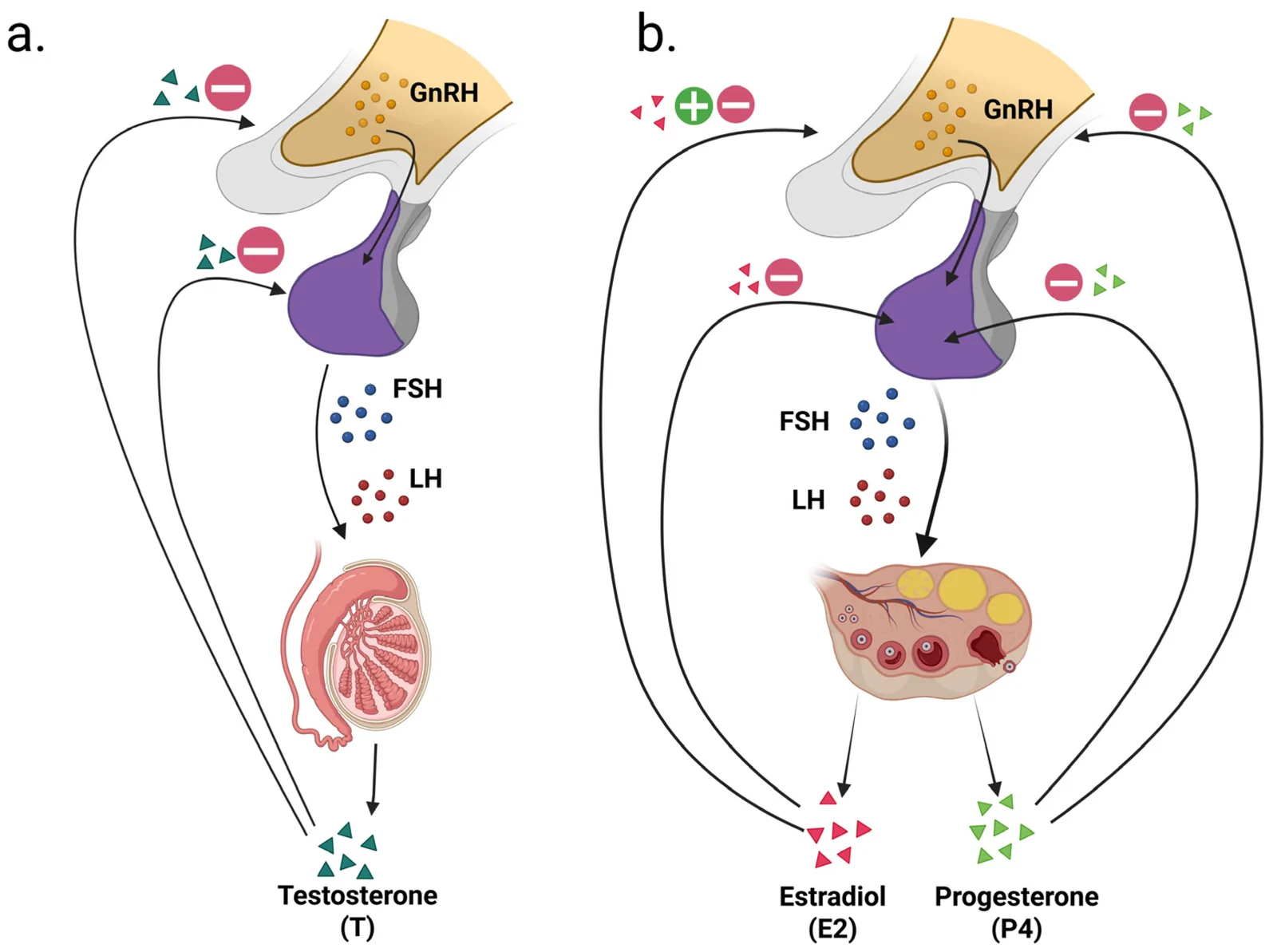
Mammalian HPG axis in (**a**) males and (**b**) females. GnRH neurons in the hypothalamus synthesize and secrete GnRH, which acts on the GnRHR located in the membrane of gonadotropes in the anterior pituitary, where it regulates the synthesis and exocytosis of gonadotropins, LH and FSH, into the general circulation. The gonadotropins act on the gonads, testes (**a**) and ovaries (**b**), regulating gametogenesis and steroidogenesis. Gonadal hormones, testosterone from the testes (**a**), and estradiol and progesterone from the ovaries (**b**) exert the regulation of their own production through feedback loops at the levels of both hypothalamus and anterior pituitary. All feedback loop mechanisms are negative, except for the estradiol feedback loop prior to ovulation, which is positive and necessary for the GnRH/LH surge. GnRH, gonadotropin-releasing hormone; FSH, follicle-stimulating hormone; LH, luteinizing hormone; T, testosterone; E2, estradiol; P4, progesterone; (−), negative feedback loop; (+), positive feedback loop.Created in BioRender. Milošević, A. (2026) https://BioRender.com/lyeyw5q, accessed on 1 August 2026.

Reproductive function is essential for species’ survival and is orchestrated by a complex interplay of neuroendocrine signals that coordinate gonadal activity and sexual behavior. Central to this regulation is the HPG axis, a hierarchical system that governs fertility through tightly controlled hormonal communication (Figure 1). At the apex, the hypothalamus secretes gonadotropin-releasing hormone (GnRH), which acts on the anterior pituitary gonadotropes by binding to the GnRH receptor (GnRHR) and stimulates the release of luteinizing hormone (LH) and follicle-stimulating hormone (FSH). These gonadotropins act on the gonads to support gametogenesis and stimulate the production of sex steroids, including testosterone, estradiol, and progesterone. In turn, the steroid hormones exert feedback effects on both the pituitary and hypothalamus, modulating the activity of the axis. The regulatory patterns of the HPG axis differ between sexes, mostly in the fact that in males, the release of neuroendocrine and endocrine factors is tonic, while in females, they are released in a cyclical manner, leading to cyclical changes in all components of the female reproductive tract, which are defined as the estrous or menstrual cycle [38].

The neurons secreting GnRH are diffusely located in the mammalian brain, due to their specific migratory route during development, extending from the olfactory tract to the mediobasal hypothalamus [39]. A defining feature of GnRH secretion is its pulsatile nature, which predominates during most of the female reproductive cycle and represents the only release pattern in males. This episodic pattern emerges late in fetal development or early postnatally, remains suppressed until puberty, and is reactivated at reproductive maturation [40]. In adult rodents, GnRH pulses occur approximately every 70 min in males and every 50 min in females [41,42]. In humans, GnRH pulse frequency is sex- and cycle-dependent, ranging from every 90–100 min in the early follicular phase to about 60 min in the late follicular phase, slowing down to 4–8 h during the luteal phase [43]. During the late follicular phase in females, GnRH release shifts from its usual pulsatile pattern to a sustained, elevated state—referred to as the GnRH surge—that persists for several hours and is critical for inducing the preovulatory LH surge and enabling ovulation in most species [44,45,46].

The central regulator of both pulsatile and surge modes of GnRH release is the network of hypothalamic kisspeptin neurons [47]. Kisspeptins, encoded by *Kiss1*, act via the G protein-coupled receptor 54 (GPR54), which are expressed in GnRH neurons [48,49]. In rodents, kisspeptin neurons are located in the anteroventral periventricular nucleus and the periventricular nucleus, as well as in the arcuate nucleus [50,51]. In rodents, the region comprising the anteroventral periventricular nucleus and the periventricular nucleus is referred to as the rostral periventricular area of the third ventricle (RP3V). The RP3V population of kisspeptin neurons is highly sexually dimorphic, with females having about ten times the number of these neurons than males [50]. Kisspeptin neurons serve as the primary interface for sex steroid feedback, since GnRH neurons themselves largely lack steroid receptors. In females, nearly all kisspeptin neurons express estrogen receptor (ER) α [52] and progesterone receptor [53], while in males they express both ERα and androgen receptors [54]. Importantly, the population of kisspeptin neurons in the arcuate nucleus serves as the regulator of the pulsatile secretion of GnRH [55,56], while the preovulatory surge of GnRH/LH in females is controlled mainly by the RP3V kisspeptin neuron population [57]. In humans, the kisspeptin neurons are located most densely in the infundibular nucleus (analogue to the arcuate nucleus), as well as in the rostral preoptic area [58].

At the pituitary level, LH and FSH are heterodimeric glycoproteins composed of a common α-subunit (encoded by the gene *Cga*) and unique β-subunits (encoded by the genes *Lhb* and *Fshb*, respectively) that confer the hormone specificity [59]. Their expression and secretion are regulated at the levels of transcription, post-translational processing, and exocytosis, under the dominant control of pulsatile GnRH [60]. GnRH acts via its receptor, expressed exclusively in gonadotropes [61]. Pulsatile GnRH maintains GnRHR expression and hormone secretion, with maximal receptor expression seen at 30-min pulse intervals in rodents [62], while continuous stimulation induces receptor downregulation [63]. Importantly, LH release is tightly coupled to GnRH pulses, whereas FSH secretion is less dependent on GnRH signaling—FSH exocytosis is constitutive [64].

**Figure 2:**
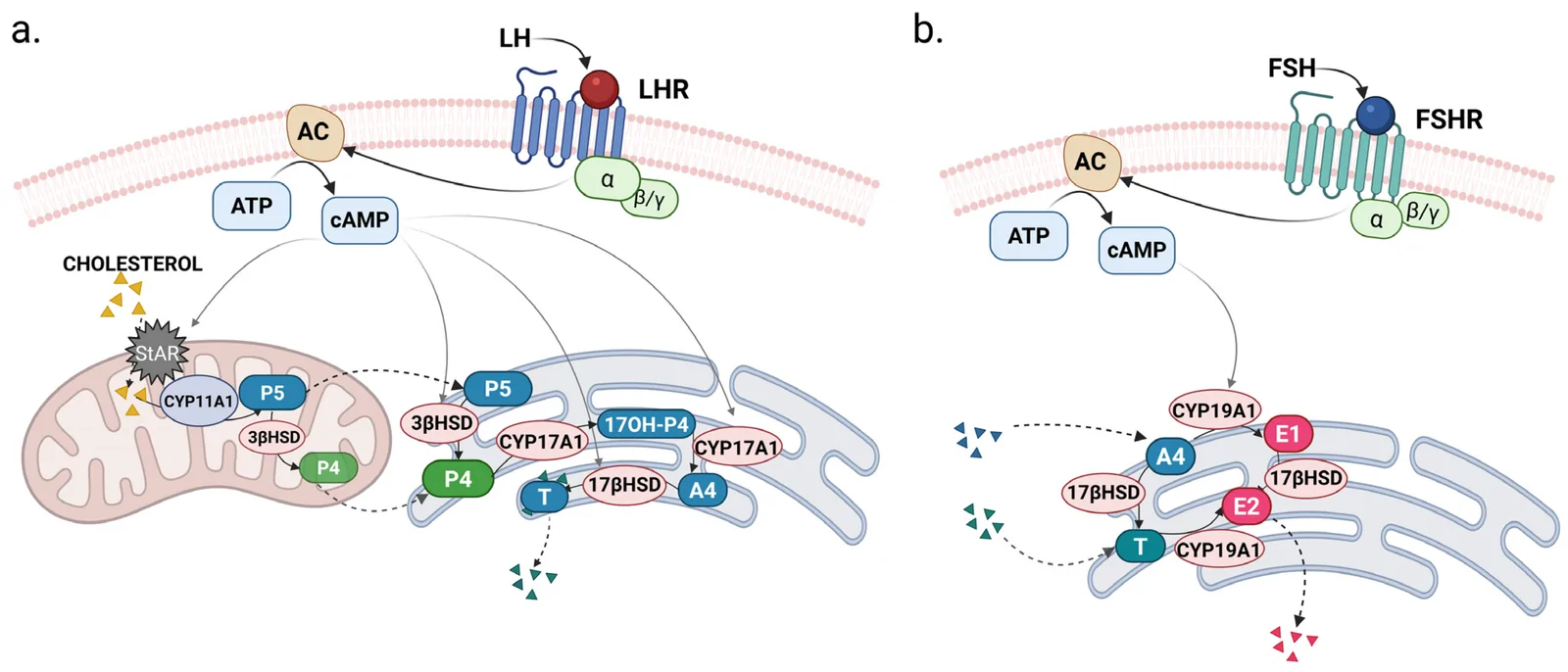
Regulation of gonadal steroidogenesis in mammals. (**a**) In Leydig cells of the testes and in the follicular theca cells in the ovaries, the synthesis of androgens takes place, regulated mostly via the LH receptor. (**b**) In ovarian follicles, the last step of steroidogenesis—the aromatization of androgens into estrogens—takes place in granulosa cells, and is regulated by the activation of FSH receptors. Abbreviations: 17α-OH-P4, 17α-hydroxyprogesterone; 17α-OH-P5, 17α-hydroxypregenenolone; 17βHSD, 17β-hydroxysteroid dehydrogenase; 3βHSD, 3β-hydroxysteroid dehydrogenase/Δ5-4 isomerase; A4, androstendione; A5, androstenediol; AC, adenylate cyclase; ATP, adenosine triphosphate; cAMP, cyclic adenosine monophosphate; CYP11A1, cytochrome P450 oxydase family 11, subfamily A, polypeptide 1; CYP17A1, 17α-hydroxylase/17,20-lyase; CYP19A1, aromatase; E1, estrone; E2, estradiol; FSH, follicle-stimulating hormone; FSHR, follicle-stimulating hormone receptor; LH, luteinizing hormone; LHR, luteinizing hormone receptor; P4, progesterone; P5, pregnenolone; StAR, steroidogenic acute regulatory protein; T, testosterone. Created in BioRender. Milošević, A. (2026) https://BioRender.com/lyeyw5q, accessed on 1 August 2026.

Gonadotropins, binding to their specific receptors on gonadal cells, trigger the cyclic adenosine monophosphate signaling cascade, coordinating the activation of the steroidogenic machinery and driving the enzymatic conversion of cholesterol into biologically active sex steroids (Figure 2). Cholesterol is supplied to cells via several pathways [65]. Steroidogenic enzymes are localized at the inner mitochondrial membrane and in the smooth endoplasmic reticulum [66]. The rate-limiting step of steroidogenesis is the transport of cholesterol across the intermembrane space from the outer to the inner mitochondrial membrane, where the first enzymatic step is taking place [67]. The transport of cholesterol, which is hormone-regulated, is mediated by the protein complex known as the transduceosome, comprising cytoplasmic and mitochondrial proteins [65], with the steroidogenic acute regulatory protein playing a central role [68]. The enzymatic machinery consists of two major families: hydroxysteroid dehydrogenases, which interconvert hydroxysteroids and ketosteroids; and cytochrome P450 oxidases, which catalyze hydroxylation and C–C bond cleavage reactions [67].

The HPG axis represents a dynamic system, responsive to internal and external cues, that ensures reproductive competence. Importantly, while the general principles in HPG axis regulation are conserved across mammalian species, there are a few species-specific differences between rodents and humans that should be considered. Overall, the ovarian cycles of the spontaneous ovulators are similar, but their duration is different—the estrous cycle in rats and mice lasts 4–5 days, while the menstrual cycle in humans lasts about 28 days and comprises a prolonged luteal phase [69]. Moreover, the neurons in the preoptic area in rodents are essential for the control of ovulation, while this region seems less important for this process in higher primates [70]. Furthermore, the LH surge in rats and mice is rigidly coupled to the circadian signal, i.e., it is regulated also by the suprachiasmatic nucleus of the hypothalamus [47], while in humans this is not the case [70]. Reproductive senescence in women, i.e., menopause, has its differences compared to that in rodents, where it is instead referred to as estropause. While both states are characterized by HPG axis dysregulation, including the decreased responsiveness of LH secretion to estrogen signaling, the main difference is that menopause includes complete ovarian failure, while potentially mature ovarian follicles can be observed in aged rodents [71].

## 2. Sex Differences in MS and EAE

Multiple sclerosis is at least twice as common in women as it is in men, and this ratio reaches 4:1 in some world regions [72]. A higher prevalence in females is also registered for several autoimmune diseases, such as systemic lupus, rheumatoid arthritis, Sjogren’s syndrome and Hashimoto thyroiditis [73].

Besides the higher prevalence, other MS parameters are sex-biased as well. For instance, women experience a more inflammatory disease phenotype, while men suffer from a higher level of neurodegeneration [74,75]. Furthermore, women are presented with more frequent relapses and an earlier onset of MS symptoms, but men are more prone to the more severe development of the disease, reflected in a higher risk for developing primary-progressive MS and greater levels of tissue damage in the CNS, as well as more pronounced cognitive dysfunctions and poorer functional connectivity [76,77,78,79]. Moreover, disease progression is faster in men than in women with RRMS, and the transition to secondary-progressive MS occurs earlier in men [80,81]. Men also show a slower recovery after relapses [77,82].

The sex bias in the incidence of EAE seems to be dependent on the strain of mice and rats and immunization protocol. For example, after the active induction of EAE, female SJL and NZW mice showed a significantly higher incidence compared to males, while no differences between sexes were observed in the B10.PL, PL/J, and C57BL6 strains [83]. Passively induced EAE in SJL/J mice is more severe when the antigen-specific cells are derived from previously immunized females, compared to males [84,85]. Interestingly, in this case, antigen selection can also affect the sex differences [86]. According to some researchers, in the Dark Agouti strain of rats, females have a higher incidence of EAE compared to males [87,88], although this was not the case in the studies done by our group [32,37].

Apart from incidence of EAE, sex differences were also observed in the progression and symptom manifestation of this disease. In the F2 generation offspring generated from SJL/J and B10.S/SgMcdJ, the two strains of mice with different susceptibility to EAE induction, the symptomatic phase lasted longer in females, with a higher cumulative disease severity, while males exhibited a higher maximal score at the peak of EAE and greater body weight loss [89]. Interestingly, while a greater degree of demyelination in the spinal cord was observed in female mice compared to males, no differences in the clinical manifestation of the disease were found [90]. In the acute model of EAE in Dark Agouti rats, females exhibit an earlier onset of the disease [32], while males experience a higher maximal score [37]. These sex-specificities, however, do not affect the cumulative disease severity, which does not differ between sexes [32,37], similar to the chronic model of EAE in the same rat strain [91]. Interestingly, it has been previously shown that the mortality rate is higher in male Dark Agouti rats compared to females in acute EAE [92]. Female Lewis rats are more prone to relapses, while males fully recover after the acute phase of EAE [93].

In mouse EAE models, pertussis toxin is typically added to induce a more robust disease onset. However, aside from inducing immunomodulatory effects that are not specific to MS, pertussis obscures the sex differences in immune responses in mice [94,95].

These data suggest that the other parameters of EAE apart from incidence, such as symptom onset and disease progression dynamics, might also be affected by the rodent strain and immunization protocol, and that these specificities should be carefully considered when modelling MS in rodents.

## 3. Sex Hormones and the Pathology of MS and EAE

The factors proposed to be driving the sex specificities in MS and EAE pathologies are the sex chromosomes and sex hormones, which are not necessarily mutually exclusive in this regard [96].

### 3.1. Sex Hormones in MS Patients

The research on the roles of sex hormones as factors influencing MS pathology is based on the fact that changes in the ratio of disease incidence between women and men are correlated with periods of fluctuations in female sex hormones, such as puberty, pregnancy or menopause.

Specifically, the ratio of girls to boys developing MS before puberty is approximately 1:1, while after puberty, the higher incidence shifts toward females [76,77]. Additionally, it has been shown that earlier menarche in girls is associated with an increased risk and earlier onset of MS symptoms [97,98,99,100]. Protective effects of pregnancy, characterized by very high levels of estrogens and progesterone, have been shown in many studies [101]. For example, pregnant MS patients show a significantly lower number of relapses [102], especially in the third trimester [103]. Additionally, women who had at least one pregnancy after the onset of MS became wheelchair-dependent later than women who had no pregnancies [104]. This effect is even more pronounced after two or more pregnancies [105], especially in patients with RRMS [106]. It is important to emphasize that parity (i.e., the number of pregnancies reaching 24 weeks), not gravidity (i.e., the total number of pregnancies), is the factor associated with protective effects in MS [79], indicating the significance of hormonal changes in the third trimester of pregnancy in particular. A significantly increased number of relapses compared to the year before pregnancy was previously registered during the three-month postpartum period [103], although more recent data do not indicate differences in relapse frequency during these two periods [102]. However, it should be noted that most women in the latter study were breastfeeding, and breastfeeding is associated with a reduced risk of relapse during the postpartum period [107]. In late-onset MS, in which the first symptoms occur after 50 years of age, when sex hormone levels are declining due to menopause, the difference in MS incidence between men and women decreases, which becomes even more pronounced in very late-onset MS, after the age of 60. On the other hand, the decrease in sex hormones during menopause may contribute to neurodegenerative changes in MS patients [108]. Supporting this are data indicating that low concentrations of anti-Müllerian hormone, indicative of follicular reserves, are associated with greater disability and gray matter loss in women with MS, independent of patient age [109].

Although the role of female sex hormone fluctuations is usually emphasized in MS pathology, some studies support the hypothesis that the reduced concentrations of androgens at various life stages in males might carry an increased risk for developing MS as well [77]. Even though no correlation was found between hormonal changes during puberty and an increased risk for MS in boys [99], the role of testosterone in MS risk might extend even into the prenatal period. Namely, the ratio of androgens to estrogens during this period is inversely reflected in the “2D:4D ratio”, i.e., the difference in length between the index finger and the ring finger, and this ratio is higher in MS patients, suggesting that lower levels of androgens in the prenatal period might affect the disease risk [110]. Additionally, MS symptoms appear later in men than in women, usually between the ages of 30 and 40, when testosterone levels begin to gradually decline, suggesting that physiological decreases in testosterone concentration during this period, also referred to as “andropause”, may contribute to the development of MS as well [111].

Interestingly, in transgender individuals, who use gender affirming cross-sex hormone treatments, an increased adjusted relative risk of MS has been reported in the case of transwomen, i.e., male-to-female transitions, compared to the control male cohort, which was not the case with transmen compared to the reference female group [112]. It has been postulated that the cause might lie in the reduction in androgen levels, or in the use of exogenous estrogens [113].

### 3.2. The Role of Sex Hormones in EAE

The importance of gonadal hormones in EAE pathogenesis is underscored by differences in disease progression observed in animals with altered gonadal status. For instance, castrated male SJL/J mice develop a relapsing form of EAE, whereas sham-operated males exhibit a monophasic disease course, both in actively induced [114] and passive EAE models [115]. In contrast, in C57BL/6 mice, no differences were found between castrated and sham-operated males, suggesting that the influence of gonadal status on EAE progression is also strain-specific [115]. The impact of gonadal status on EAE progression is also strain-dependent in rats: castration of male Lewis rats has no effect on EAE incidence or severity [116], whereas in Wistar rats, castration leads to longer duration of the disease [117].

Another example of the importance of sex hormones for the course of EAE are the physiological fluctuations in their levels, which play a role in the development of the disease. Similar to the protective effect seen in the third trimester of pregnancy in women with MS, female mice with EAE exhibit a marked reduction in relapse frequency during the second half of gestation, followed by increased disease activity postpartum [118]. Additionally, when gravid female mice are immunized during the second or third week of gestation, there is a decrease in EAE incidence, fewer relapses, and delayed symptom onset, whereas postpartum immunization is associated with worsened relapses [118,119]. In rats, even immunization during early gestation leads to reduced cumulative disease severity and maximal clinical scores [120], or to delayed symptom onset or complete absence of symptoms [121]. However, one study found greater clinical improvement when the animals were immunized during the third week compared to the first two weeks of gestation [122], further supporting the observed protective effects of late pregnancy in MS patients. The protective mechanisms of pregnancy in EAE include reduced immune cell proliferation [118,123], increased production of anti-inflammatory cytokines, and decreased production of pro-inflammatory cytokines [119]. Moreover, progesterone binding to glucocorticoid receptors on T cells directs their differentiation toward a regulatory T cell phenotype [124], which may also contribute to the immunomodulatory effects of pregnancy in EAE. Since the importance of sex hormones for MS pathology is also shown in menopausal women, this stage in the reproductive life should be considered in animal models of MS as well. As explained above, there are important differences in the female reproductive senescence between rodents and humans, which is why faithfully modelling menopausal transition has its challenges. Ovariectomy is most commonly used as a surgical model of menopause, most closely representing the depletion in gonadal hormone levels and ovarian reserve failure experienced in menopausal women [71]. However, the literature data on the effects of ovariectomy on EAE progression is diverse. For example, ovariectomy in B10.RIII mice leads to earlier symptom onset [125], while in SJL/J × B10.S hybrids, it is associated with shorter disease duration and higher clinical scores [89]. On the other hand, in C57BL/6 mice, either no significant differences between sham-operated and ovariectomized females were reported [126,127], or ovariectomy exacerbated EAE symptoms [128]. It is important to highlight that, together with the difference in strains, the immunization protocols and the age at which the animals were ovariectomized in the reported studies also varied, adding another level of complexity when modelling MS in menopause-mimicking conditions in rodents.

### 3.3. Beyond Sex Hormones

Given the marked female predominance of MS, EAE studies increasingly include both sexes to distinguish biological sex differences from experimental variability. However, accumulating evidence indicates that sex differences in CNS autoimmunity extend beyond the actions of estrogen and testosterone. The four-core genotype mouse model, in which gonadal sex is uncoupled from the sex chromosome complement, demonstrated that the XX chromosome complement increases susceptibility to EAE, whereas the XY complement promotes greater neurodegeneration in the SJL strain [129,130]. Furthermore, sex-biased immune responses are increasingly recognized to arise from interactions between sex chromosomes, gonadal hormones, and immune-cell intrinsic regulatory pathways rather than from circulating sex steroids alone [131]. Consistent with this concept, a recent study showed that the efficacy of TNFR2-targeted therapy depends on both sex chromosome complements and gonadal production of activin A, identifying a non-steroidal gonadal pathway that shapes neuroinflammation and therapeutic responses independently of classical sex steroids [132].

Together, these findings demonstrate that biological sex is a multidimensional variable, with sex chromosomes, steroid hormones, non-steroidal gonadal factors, and immune-cell intrinsic mechanisms all contributing to disease susceptibility and therapeutic outcome.

## 4. HPG Axis Dysregulation in MS/EAE

The relationship between sex hormone levels and the course of MS/EAE is bidirectional—apart from the influence of hormonal fluctuations on the disease risk and symptom occurrence, the course of MS/EAE itself may lead to disturbances in HPG axis function and the changes in sex hormone levels.

### 4.1. Changes in the Function of the HPG Axis in Patients with MS

For women, the phase of the menstrual cycle seems to be important for the pathology of MS. In this regard, premenstrual worsening of the symptoms was recorded in over 40% of female MS patients [133,134]. Moreover, a significant reduction in estradiol concentration in patients with MS was recorded in the luteal, but not in the follicular phase [135], while other researchers did not detect changes in the concentration of this hormone in either of the menstrual cycle phases [136]. In contrast, other researchers did not observe changes in estradiol concentration in the luteal phase, but rather a decrease in this hormone during ovulation in patients with MS [137,138]. In addition, reduced estradiol concentrations are correlated with a higher frequency of relapses in patients with MS [139], further highlighting the importance of this hormone for MS pathology. When it comes to progesterone, no differences in its concentration were observed in female MS patients, compared with healthy women [135,136,137,138]. On the other hand, in men with MS, progesterone concentration is significantly increased [140]. Interestingly, the ratio between progesterone and estradiol concentrations may be associated with disease activity in female patients with MS. However, the literature does not offer consistent findings: high estradiol with low progesterone has been associated with greater MS activity [141], but increased disease activity has also been correlated with a higher progesterone-to-estradiol level ratio during the luteal phase of the menstrual cycle in MS patients [142].

Some literature data indicate that testosterone levels in patients with MS do not change [135,143]. However, multiple studies have shown that circulating testosterone levels are significantly reduced [31,144,145], which correlates with a greater degree of disability in men with MS [146]. It is important to note that in several studies reporting low testosterone levels in MS patients, LH levels were not elevated [31,145,146]. In addition, lower testosterone levels have also been measured in women with MS [135,136], both in the follicular and the luteal phase of the menstrual cycle [137]. In contrast, earlier studies have shown that female MS patients have elevated testosterone levels compared to healthy women, and that this increase is even higher in patients with higher disability scores [147].

Overall, the amount of data on the changes in sex hormone levels in patients of both sexes is limited. Together with this, the fact that some studies show contrasting results and, in many cases, the lack of accounting for the menstrual cycle status in female MS patients greatly contribute to the need for a more in-depth and systematic approach in exploring the function of reproductive status in people suffering from MS, as well as other neuroinflammatory and neurodegenerative diseases.

### 4.2. Dysregulation of the HPG Axis Following the Course of EAE

Even though EAE has been extensively used to mimic many features of MS, few studies have systematically addressed changes in the functionality of the HPG axis in EAE and other MS models. However, the literature does offer some data on a few aspects of changes in the HPG axis in this context.

One of the first observable changes in the functioning of the HPG axis in females is the change in the dynamics of the estrous cycle. For example, an active induction of EAE in C57BL/6 mice leads to an arrest in the diestrus phase and to the resumption of cyclicity two weeks after immunization [148], which is similar to the transient arrest in diestrus phase concomitant with the symptomatic phase of acute EAE in Dark Agouti rats, which our laboratory has observed [32,34]. On the other hand, in SJL mice, EAE causes shortening of the estrous cycle at the expense of diestrus [149]. A transient arrest in diestrus was also observed in the cuprizone model of demyelination [150].

Changes in the dynamics of the estrous cycle also point to changes in sex hormone levels, and furthermore, to changes in the regulation systems of their synthesis. Pertinent to this, reduced progesterone concentration was identified in both acute [151] and chronic models of EAE in female rats [152], which is the opposite of our findings that circulating progesterone levels are increased both in female and male rats in the acute model of EAE [32,34,37]. Estradiol concentration remains unchanged in female mice with passively induced EAE [31], as well as in Dark Agouti rats following active immunization [32].

Significantly reduced circulating testosterone levels were reported in male DA rats during the symptomatic phase of EAE [32,33], as well as in mice after passive induction of the disease [31]. However, the latter study reported elevated LH levels together with decreased testosterone levels, suggesting that testes are the primary origin of the hypogonadal-like changes [31]. In contrast, our group has shown the opposite—circulating LH levels are decreased and correlated with low testosterone levels in males [32,33]. Interestingly, we have also found increased testosterone levels in female rats during the symptomatic phase of EAE [34].

**Figure 3:**
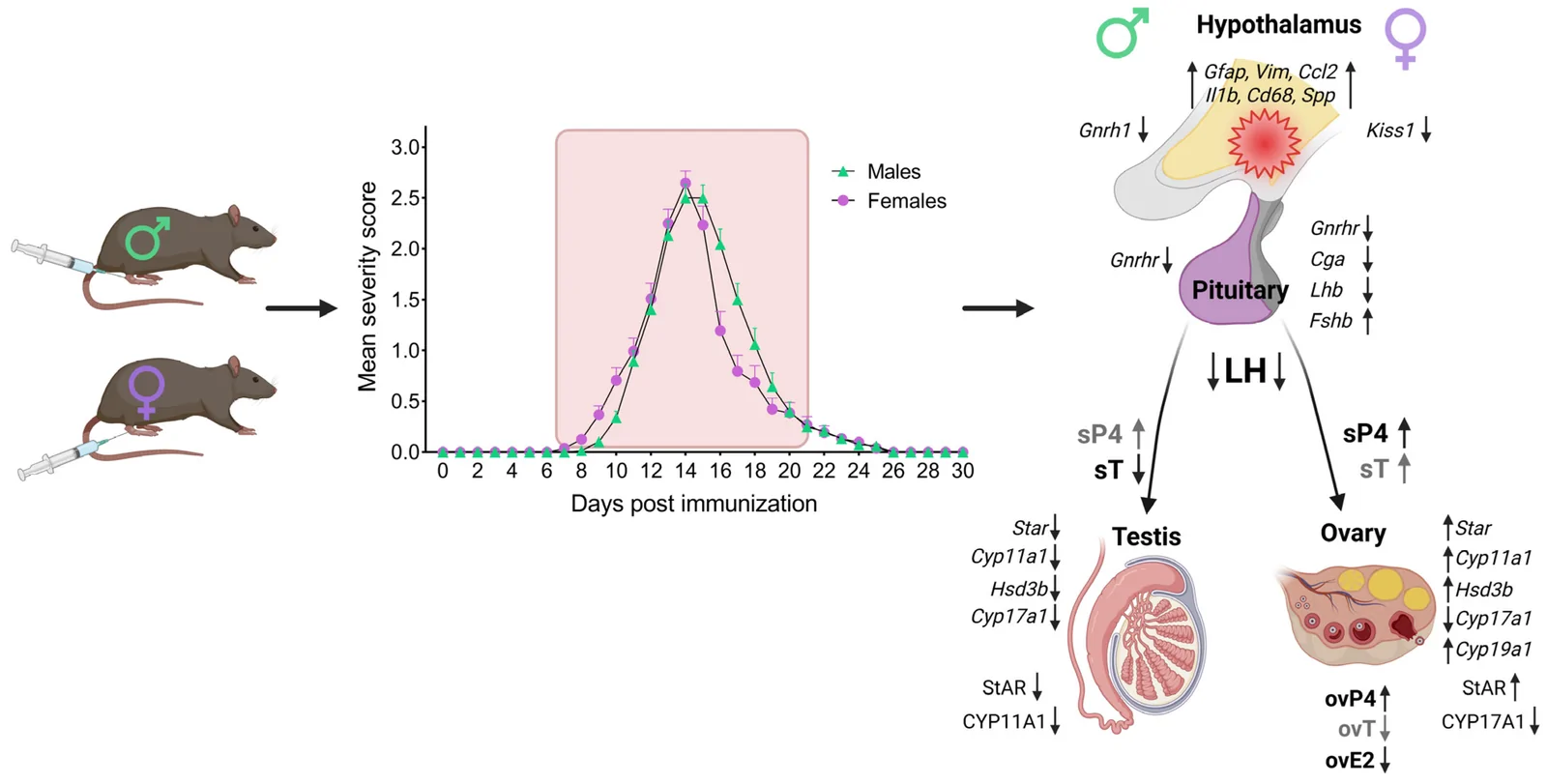
Overview of changes in the HPG axis in an acute EAE model in rats of both sexes. Active immunization leads to an acute monophasic course of EAE in Dark Agouti rats of both sexes, causing a transient arrest in reproductive capacity in both males and females. The individual changes along the HPG axis, at the level of each of its components, are sex specific. However, common characteristics of these changes are the decrease in circulating LH levels and the inflammation in the hypothalamus, which seems to play a role as the origin of all downstream changes. The line graph is taken from Milosevic et al., 2024 [37], while the changes along the HPG axis are an illustration of compiled data from several studies performed by our group [32,33,34,37]. Abbreviations: CYP11A1, cytochrome P450 oxydase family 11, subfamily A, polypeptide 1; CYP17A1, 17α-hydroxylase/17,20-lyase; ovP4, ovarian progesterone; ovT, ovarian testosterone; sP4, serum progesterone; sT, serum testosterone; StAR, steroidogenic acute regulatory protein. Created in BioRender. Milošević, A. (2026) https://BioRender.com/lyeyw5q, accessed on 1 August 2026.

Moreover, apart from the measurement of sex hormone levels described above, our laboratory has shown for the first time a systemic analysis of the changes in the HPG axis functions during EAE in animals of both sexes. We have shown that circulating LH levels are decreased in females as well, and that this decrease in LH levels in both males and females follows changes in the expression of the hypothalamic and pituitary genes responsible for the regulation of the HPG axis, which are sex-specific [32]. This arrest in reproductive capacity, observed in both males and females, is reflected in a sex-specific manner at the level of gonads as well. Namely, testicular steroidogenic machinery is completely downregulated at the gene and/or protein level but remains maximally responsive to the exogenous stimulation of LH receptor [33]. In contrast, ovarian steroidogenic components are mostly upregulated, except for the androgen-producing enzyme CYP17A1, leading to an increase in ovarian progesterone content, and a decrease in ovarian testosterone and estradiol, causing a pseudopregnancy-like state [34]. Importantly, despite the sex-specificities, the common hallmark of this state in both sexes, besides the decrease in circulating LH levels, is inflammation in the hypothalamus, registered both in males and females [32], which could be viewed as the culprit of all the downstream changes. It is also important to underscore that the observed changes are reversible and limited to the symptomatic phase of EAE. The findings described here are summarized in Figure 3.

Another common feature in rats of both sexes are the elevated serum progesterone levels that, interestingly, stem from different sources: in males, the adrenal glands, not the testes, are responsible for this increase [37], while in females this elevation originates in the ovaries [34].

### 4.3. Limitations of Translation

EAE is an experimental model developed to investigate specific mechanisms of CNS autoimmunity rather than to fully recapitulate the complexity of human MS. More broadly, translation from animal models to humans is limited by the use of genetically homogeneous animals maintained under highly controlled laboratory conditions, which do not capture the genetic, environmental, and lifestyle heterogeneity of human populations. In contrast to experimental settings, MS susceptibility and progression are shaped by complex interactions between genetic background and environmental factors, including vitamin D levels, Epstein–Barr virus infection, smoking, obesity, diet, and other lifestyle-related exposures that cannot be faithfully modeled in laboratory animals.

Although the organization of the HPA and HPG axes is highly conserved across mammals, important species-specific differences should be considered when extrapolating findings from rodents to humans. Humans primarily secrete cortisol, whereas rodents predominantly produce corticosterone. Although these glucocorticoids signal through the same receptors, they differ in pharmacokinetics, metabolism, receptor affinity, and tissue distribution, potentially influencing neuroendocrine regulation of immune responses [153,154]. Furthermore, humans are diurnal, whereas rodents are nocturnal, resulting in opposite circadian patterns of glucocorticoid secretion. Because circadian clock genes regulate both immune function and endocrine signaling, these differences may contribute to distinct inflammatory and immunomodulatory responses in EAE and MS [155,156,157].

The HPG axis also exhibits important species-specific characteristics. While its fundamental organization and hormonal regulation are conserved, female rodents undergo a short estrous cycle, whereas women exhibit a menstrual cycle characterized by menstruation and extensive endometrial remodeling. In addition, differences in hypothalamic organization, reproductive aging, and the temporal dynamics of sex steroid secretion may influence immune regulation and responses to hormonal interventions.

Collectively, these limitations should not be viewed as shortcomings of animal models but rather as important considerations when interpreting preclinical findings. Integrating evidence from complementary experimental models with findings from well-designed clinical studies remains essential for translating mechanistic insights into improved understanding and treatment of MS.

## 5. Therapeutic Potential of Manipulating the HPG Axis in MS and EAE

Over the years, research in EAE, but also in other models of MS, has given rise to many therapeutic approaches that mostly target the immune component of the MS course or the glia cells [158]. Given the strong evidence that the changes in the incidence, course, and symptom manifestation of MS/EAE are at least in part influenced by sex hormones, the effects of exogenous administration of sex hormones and their derivatives on these pathologies have been explored as well.

For example, pretreatment with estradiol leads to a reduced incidence of EAE, a delayed onset of symptoms, and lower overall disease severity in mice of both sexes, and adoptive transfer of encephalitogenic T lymphocytes isolated from those pretreated donors is significantly less effective in inducing EAE [159]. Similarly, administration of ethinyl estradiol delays the onset of EAE and reduces symptom severity, accompanied by decreased infiltration of immune cells into CNS tissue [160]. The demonstrated protective effects of estradiol and its derivatives are likely mediated through a reduction in levels of pro-inflammatory cytokines characteristic of Th1 lymphocytes, but without an increase in anti-inflammatory cytokine levels [161,162]. Interestingly, this immunomodulatory action of estradiol in EAE most likely occurs via ERα [163,164], whereas both ERs seem to play a role in its neuroprotective actions [164]. In MS patients, administration of ethinyl estradiol via oral contraceptives showed positive, dose-dependent effects, including a reduction in the size of active inflammatory lesions and improvement in cognitive functions [165,166].

Pretreatment with estriol alleviates EAE symptoms and/or leads to their delayed onset in male mice of various strains [159,167]. In female C57BL/6 mice, pretreatment with estriol completely prevents disease development [167]. Prior gonadal status of females seems irrelevant for the effects of estriol, as this treatment delays symptom onset by up to 25 days even in ovariectomized females [125], and results in significantly milder symptoms and an almost complete absence of demyelinating and inflammatory lesions [168]. High doses of estriol given to MS patients lead to a reduction in IFN-γ in unstimulated peripheral blood mononuclear cells [169]. A decrease in pro-inflammatory and an increase in anti-inflammatory cytokine levels following estriol administration were also confirmed in stimulated peripheral blood mononuclear cells [170]. These results were correlated with a reduction in lesion size observed on magnetic resonance imaging. The effects of estriol in these studies were more pronounced in patients with RRMS compared to those with secondary-progressive MS [169,170]. In women with relapsing-remitting MS, estriol leads to a reduced relapse rate and improvement in cognitive functions [171], which was also confirmed by a reduction in gray matter atrophy [172].

The protective effects of progesterone observed in EAE may be a consequence of reduced levels of pro-inflammatory cytokines [173,174]. However, the protective action of this hormone is also reflected in the prevention of axonal damage [175], as well as through direct effects on oligodendrocytes and the promotion of remyelination [176,177]. Pretreatment with progesterone leads to an improvement in the clinical parameters of EAE, accompanied by reduced immune cell infiltration and demyelination in the spinal cord of female mice [178], and an even more pronounced improvement was observed when the effects of a combination of progesterone and estradiol were examined [179]. In contrast, progesterone treatment in ovariectomized female rats resulted in increased immune cell infiltration in the spinal cord [180], indicating that the gonadal status of females is also important for the effects of progesterone in the EAE model. The use of progesterone derivatives in patients with MS was attempted in combination with estradiol in order to prevent relapses during the postpartum period, but this study was discontinued due to an insufficient patient response [181].

One of the factors that might drive gender biases in MS/EAE pathologies is the protective effect of androgens. The fact that the androgen receptor, binding both testosterone and dihydrotestosterone, is expressed in many cell types including immune cells, has led to the general view of androgens as protective factors in autoimmune diseases [182]. Indeed, treatments with androgenic hormones in animal models of MS exert immunomodulatory, neuroprotective, and promyelinating effects [80]. The immunomodulatory action of androgens is achieved through reduced production of pro-inflammatory and increased production of anti-inflammatory cytokines in immune cells, both after in vitro treatment [159], and following in vivo treatment [183,184]. When encephalitogenic T lymphocytes treated with androgens are used for passive induction of EAE, they induce a milder disease [85]. Oral administration of testosterone in male Wistar rats leads to a reduced incidence of EAE, and in castrated animals it mitigates the negative effects of castration on symptom duration [185]. In addition to effects on cytokine production, treatment with dihydrotestosterone also reduces the expression of neuroinflammation markers in the spinal cord, alleviating disease symptoms, as shown in a chronic EAE model in Dark Agouti rats [186]. Furthermore, treatment with this hormone has been shown to improve synaptic transmission and preserve hippocampal functionality in mice with EAE [187]. In the cuprizone model of demyelination, testosterone and estradiol were shown to have promyelinating effects, whereas dihydrotestosterone did not, which may indicate that testosterone in this context acts via ERs after its conversion to estradiol [188]. However, it has also been demonstrated that, at the site of a demyelinating lesion in the spinal cord induced by lysolecithin in mice, testosterone binds to androgen receptors expressed on astrocytes, activates them and recruits oligodendrocytes, leading to remyelination [189]. A functional androgen receptor in the corpus callosum is required for the promyelinating action of testosterone, as well as of its derivatives that cannot be aromatized [190].

Daily administration of a gel containing 100 mg of testosterone for one year in 10 patients with RRMS led to improvements in cognitive abilities and a significant reduction in brain atrophy, although no change in the number or size of inflammatory lesions was observed [191]. Patients from the same trial were also followed for six months after discontinuation of therapy, and magnetic resonance imaging showed that the extent of gray matter loss after testosterone therapy was significantly reduced compared to the baseline, measured at the start of the trial [192]. Testosterone administered in the same manner leads to a reduction in the number of CD4^+^ T lymphocytes and an increase in the NK cell population, as well as a decrease in levels of the pro-inflammatory cytokine IL-2 and an increase in levels of various growth factors in peripheral blood mononuclear cells [193]. The use of testosterone and its derivatives as therapeutics for MS remains an active area of research—a clinical trial investigating the neuroprotective and remyelinating effects of testosterone undecanoate in patients with RRMS is still recruiting participants (https://clinicaltrials.gov/study/NCT03910738, accessed on 22 June 2026) [194]. However, the evidence regarding the effects of androgens in female MS patients is scarce, and this limitation should be considered when drawing conclusions on the use of androgens as potential therapeutics. Pertinent to this, the use of androgen-modifying therapies in older male patients with MS was recently observed to, unexpectedly, have potentially exacerbating effects on the disease activity [195], suggesting that the use of androgen-modifying therapies should be carefully administered while considering age- and sex-related factors.

A comprehensive list of the main findings on the use of sex hormones as potential therapeutics in the pathology of MS/EAE, as described above, is shown in Table 1.

Importantly, the systemic analysis of the changes in the functionality of the entire HPG axis during EAE in animals of both sexes, as described above, suggests that, apart from the exogenous administration of sex hormones, the potential for treating MS patients lies within promoting their endogenous synthesis. However, this approach highlights the need to evaluate changes in the HPG axis as a whole, together with its intricate connections with other neuroendocrine and endocrine systems, in each patient with MS.

## 6. Conclusions

The research in MS and its animal models, most commonly EAE, in the past decades has yielded many therapeutics treating various aspects of the disease. Nevertheless, there is still a significant need for continued research in MS, especially because its incidence has risen about 30% in all world regions [72]. The role of sex hormones in the development of MS and as potential therapeutics remains an active area of discussion [113,196], highlighting their importance. While sex hormones have demonstrated neuroprotective potential and yielded promising outcomes in preclinical and clinical MS research, their implementation in patient care is limited by the complexity of the hormonal profile of each patient and concerns regarding other potential health risks. Systemic analysis of changes along the entire HPG axis in rats of both sexes during EAE, as described above, suggests that the use of factors regulating the synthesis of gonadal steroids to promote their endogenous secretion might be a promising path in the search for adjuvant therapeutics for MS. However, the limitations of the model should also be considered, as all animal models of MS have certain differences compared to its pathophysiology. Nonetheless, EAE remains the most prominent animal model of MS, representing most of the aspects of the disease, which is the reason it is still widely used.

In conclusion, taking into account all aspects of the HPG axis function and its complex interactions with other physiological systems, together with regular monitoring of hormonal status in patients with MS, may provide a foundation for improved disease management using existing therapies, as well as support the development of novel therapeutic strategies.

## Figures and Tables

**Table 1:** The main findings on the use of sex hormones as therapeutics in MS/EAE.

Hormone Class	Hormone	Disease/Model	Species	Sex	Main Findings	Reference
Estrogens	Ethinyl estradiol	Relapsing EAE	SJL/J mice	F	Ethinyl estradiol prevented/treated EAE, reducing CNS inflammatory-cell infiltration and clinical scores.	[160]
	17β-Estradiol	Relapsing EAE	SJL/J mice	F	Low-dose estradiol significantly reduced clinical severity and delayed disease onset.	[159]
	17β-Estradiol	EAE	C57BL/6 mice	F	Estradiol reduced disease severity and decreased TNF-α production.	[161]
	17β-Estradiol	Relapsing EAE	SJL/J mice	F	Estradiol reduced CNS expression of pro-inflammatory cytokines.	[162]
	17β-Estradiol	Relapsing EAE	B10.PL mice	F	The protective effect of estradiol requires ERα.	[163]
	ERα agonist/ERβ agonist	Chronic EAE	C57BL/6 mice	F	ERα activation primarily suppressed inflammation; ERβ activation promoted neuroprotection and axonal preservation.	[164]
	Estriol	Relapsing EAE	SJL/J mice	M	Estriol reduced EAE severity in male mice.	[167]
	Estriol	Relapsing EAE	SJL/J mice	F	Estriol reduced clinical severity of EAE	[168]
	Estriol	RRMS	Human	F	Estriol treatment reduced MRI lesions and improved immunological profile.	[169]
	Estriol	RRMS	Human	F	Estriol increased production of anti-inflammatory cytokines IL-5 and IL-10, while reducing TNF-α production.	[170]
	Estriol	RRMS	Human	F	Lower relapse rate and improved inflammatory outcomes when estriol was combined with glatiramer acetate.	[171]
	Estriol	RRMS	Human	F	Preservation of cortical gray matter during estriol treatment.	[172]
Progestogens	Progesterone	Chronic EAE	C57BL/6 mice	F	Progesterone reduced clinical severity, demyelination, axonal injury and neuroinflammation while promoting oligodendrocyte survival.	[173]
	Progesterone	Chronic EAE	C57BL/6 mice	F	Progesterone significantly reduced disease severity and increased IL-10 production.	[174]
	Progesterone	Chronic EAE	C57BL/6 mice	F	Progesterone preserved axonal integrity and reduced spinal cord pathology independently of effects on inflammation.	[175]
	Progesterone	EAE	Lewis rats	F	Progesterone promoted neuronal preservation during EAE.	[180]
Progestogens + Estrogens	Progesterone + 17β-estradiol	Chronic EAE	C57BL/6 mice	F	Combined hormone therapy prevented EAE development more effectively than either hormone alone.	[179]
	Combined oral contraceptives	RRMS	Human	F	Estroprogestins combined with interferon-β improved cognitive outcomes compared with interferon-β only.	[165]
	Combined oral contraceptives	RRMS	Human	F	Oral contraceptives combined with interferon-β reduced MRI disease activity.	[166]
Androgens	Testosterone	Relapsing EAE	SJL/J mice	M	Testosterone therapy ameliorated EAE and shifted T-cells toward a Th2 phenotype.	[183]
	Testosterone	Passive EAE	SJL/J mice	M	Testosterone stimulated production of IL-10 by CD4^+^ T cells.	[184]
	Testosterone	Acute EAE	Wistar rats	M	Oral testosterone reduced disease severity and delayed disease progression.	[185]
	Dihydrotestosterone	Chronic EAE	C57BL/6 mice	F	Dihydrotestosterone reduced neuroinflammation, demyelination and axonal degeneration.	[186]
	Testosterone	Chronic EAE	C57BL/6 mice	M	Testosterone preserved hippocampal excitatory synaptic transmission during EAE.	[187]
	Testosterone	RRMS	Human	M	Testosterone treatment slowed brain atrophy, improved cognitive performance and had immunomodulatory effects.	[191]
	Testosterone	RRMS	Human	M	MRI analyses demonstrated preservation of gray matter volume during testosterone therapy.	[192]
	Testosterone	RRMS	Human	M	Testosterone increased production of neurotrophic factors and induced an anti-inflammatory immune profile.	[193]

Abbreviations: EAE—experimental autoimmune encephalomyelitis; RRMS—relapsing-remitting multiple sclerosis; F—female; M—male; CNS—central nervous system; TNF-α—tumor necrosis factor α; ERα—estrogen receptor α; ERβ—estrogen receptor β; MRI—magnetic resonance imaging; IL-5—interleukin-5; IL-10—interleukin-10.

## Data Availability

No new data were created or analyzed in this study. Data sharing is not applicable to this article.

## Abbreviations

The following abbreviations are used in this manuscript:

CNS	Central nervous system
EAE	Experimental autoimmune encephalomyelitis
ER	Estrogen receptor
FSH	Follicle-stimulating hormone
GnRH	Gonadotropin-releasing hormone
GnRHR	Gonadotropin-releasing hormone receptor
HPG	Hypothalamic–pituitary–gonadal
LH	Luteinizing hormone
MS	Multiple sclerosis
RP3V	Rostral periventricular area of the 3rd ventricle

## References

[1] Khan G., Hashim M.J. (2025). Epidemiology of Multiple Sclerosis: Global, Regional, National and Sub-National-Level Estimates and Future Projections. J. Epidemiol. Glob. Health.

[2] Atlas of MS. https://atlasofms.org/map/global/epidemiology/number-of-people-with-ms.

[3] Lassmann H. (2018). Multiple Sclerosis Pathology. Cold Spring Harb. Perspect. Med..

[4] Axisa P.P., Hafler D.A. (2016). Multiple sclerosis: Genetics, biomarkers, treatments. Curr. Opin. Neurol..

[5] Reich D.S., Lucchinetti C.F., Calabresi P.A. (2018). Multiple Sclerosis. N. Engl. J. Med..

[6] Bjornevik K., Cortese M., Healy B.C., Kuhle J., Mina M.J., Leng Y., Elledge S.J., Niebuhr D.W., Scher A.I., Munger K.L. (2022). Longitudinal analysis reveals high prevalence of Epstein-Barr virus associated with multiple sclerosis. Science.

[7] Lublin F.D., Reingold S.C., Cohen J.A., Cutter G.R., Sørensen P.S., Thompson A.J., Wolinsky J.S., Balcer L.J., Banwell B., Barkhof F. (2014). Defining the clinical course of multiple sclerosis: The 2013 revisions. Neurology.

[8] Montalban X., Lebrun-Frénay C., Oh J., Arrambide G., Moccia M., Pia Amato M., Amezcua L., Banwell B., Bar-Or A., Barkhof F. (2025). Diagnosis of multiple sclerosis: 2024 revisions of the McDonald criteria. Lancet Neurol..

[9] Compston A., Coles A. (2008). Multiple sclerosis. Lancet.

[10] Noseworthy J.H., Lucchinetti C., Rodriguez M., Weinshenker B.G. (2000). Multiple sclerosis. N. Engl. J. Med..

[11] Segal B.M. (2019). The Diversity of Encephalitogenic CD4+ T Cells in Multiple Sclerosis and Its Animal Models. J. Clin. Med..

[12] Glatigny S., Bettelli E. (2018). Experimental Autoimmune Encephalomyelitis (EAE) as Animal Models of Multiple Sclerosis (MS). Cold Spring Harb. Perspect. Med..

[13] Lassmann H., Bradl M. (2017). Multiple sclerosis: Experimental models and reality. Acta Neuropathol..

[14] Wootla B., Eriguchi M., Rodriguez M. (2012). Is multiple sclerosis an autoimmune disease?. Autoimmune Dis..

[15] ’t Hart B.A., Luchicchi A., Schenk G.J., Stys P.K., Geurts J.J.G. (2021). Mechanistic underpinning of an inside-out concept for autoimmunity in multiple sclerosis. Ann. Clin. Transl. Neurol..

[16] Titus H.E., Chen Y., Podojil J.R., Robinson A.P., Balabanov R., Popko B., Miller S.D. (2020). Pre-clinical and Clinical Implications of “Inside-Out” vs. “Outside-In” Paradigms in Multiple Sclerosis Etiopathogenesis. Front. Cell Neurosci..

[17] Stys P.K., Zamponi G.W., van Minnen J., Geurts J.J. (2012). Will the real multiple sclerosis please stand up?. Nat. Rev. Neurosci..

[18] Sen M.K., Almuslehi M.S.M., Shortland P.J., Coorssen J.R., Mahns D.A. (2020). Revisiting the Pathoetiology of Multiple Sclerosis: Has the Tail Been Wagging the Mouse?. Front. Immunol..

[19] Constantinescu C.S., Farooqi N., O’Brien K., Gran B. (2011). Experimental autoimmune encephalomyelitis (EAE) as a model for multiple sclerosis (MS). Br. J. Pharmacol..

[20] Batoulis H., Recks M.S., Addicks K., Kuerten S. (2011). Experimental autoimmune encephalomyelitis--achievements and prospective advances. Apmis.

[21] Karpus W.J. (2020). Cytokines and Chemokines in the Pathogenesis of Experimental Autoimmune Encephalomyelitis. J. Immunol..

[22] Van Kaer L., Postoak J.L., Wang C., Yang G., Wu L. (2019). Innate, innate-like and adaptive lymphocytes in the pathogenesis of MS and EAE. Cell Mol. Immunol..

[23] Brambilla R. (2019). The contribution of astrocytes to the neuroinflammatory response in multiple sclerosis and experimental autoimmune encephalomyelitis. Acta Neuropathol..

[24] Chu F., Shi M., Zheng C., Shen D., Zhu J., Zheng X., Cui L. (2018). The roles of macrophages and microglia in multiple sclerosis and experimental autoimmune encephalomyelitis. J. Neuroimmunol..

[25] O’Loughlin E., Madore C., Lassmann H., Butovsky O. (2018). Microglial Phenotypes and Functions in Multiple Sclerosis. Cold Spring Harb. Perspect. Med..

[26] Ransohoff R.M. (2012). Animal models of multiple sclerosis: The good, the bad and the bottom line. Nat. Neurosci..

[27] Nalepa I.F., Haahr N.O., Bekiaris V., Jakobsen M.R., Asuni A.A., Benmamar-Badel A. (2026). Insights and challenges on animal models for progressive multiple sclerosis. J. Neuroinflammation.

[28] Baker D., Amor S. (2014). Experimental autoimmune encephalomyelitis is a good model of multiple sclerosis if used wisely. Mult. Scler. Relat. Disord..

[29] Reyes-Reyes E.M., Chinnasamy D., Trial M.D., Fernandez F., Nguyen V.D., He Q., Leslie A.G., Begay J., Triplett J., Bradford D. (2026). HLA-DRB1*15:01 drives sex- and age-dependent microglial immune phenotypes and neuroimmune signaling. Front. Immunol..

[30] Karbalaee M., Lin A., Peruzzotti-Jametti L., Pluchino S., Mozafari S. (2026). From insights to innovations: Evaluating preclinical paradigms in demyelinating disease therapeutics. Lab. Anim..

[31] Foster S.C., Daniels C., Bourdette D.N., Bebo B.F. (2003). Dysregulation of the hypothalamic-pituitary-gonadal axis in experimental autoimmune encephalomyelitis and multiple sclerosis. J. Neuroimmunol..

[32] Milosevic A., Janjic M.M., Lavrnja I., Savic D., Bozic I.D., Tesovic K., Jakovljevic M., Pekovic S., Stojilkovic S.S., Bjelobaba I. (2020). The sex-specific patterns of changes in hypothalamic-pituitary-gonadal axis during experimental autoimmune encephalomyelitis. Brain Behav. Immun..

[33] Milosevic A., Bjelobaba I., Bozic I.D., Lavrnja I., Savic D., Tesovic K., Jakovljevic M., Stojilkovic S.S., Janjic M.M. (2021). Testicular steroidogenesis is suppressed during experimental autoimmune encephalomyelitis in rats. Sci. Rep..

[34] Milosevic A., Lavrnja I., Savic D., Milosevic K., Skuljec J., Bjelobaba I., Janjic M.M. (2023). Rat Ovarian Function Is Impaired during Experimental Autoimmune Encephalomyelitis. Cells.

[35] Heesen C., Gold S.M., Huitinga I., Reul J.M. (2007). Stress and hypothalamic-pituitary-adrenal axis function in experimental autoimmune encephalomyelitis and multiple sclerosis—A review. Psychoneuroendocrinology.

[36] Trifunovic S., Stevanovic I., Milosevic A., Ristic N., Janjic M., Bjelobaba I., Savic D., Bozic I., Jakovljevic M., Tesovic K. (2021). The Function of the Hypothalamic-Pituitary-Adrenal Axis During Experimental Autoimmune Encephalomyelitis: Involvement of Oxidative Stress Mediators. Front. Neurosci..

[37] Milosevic A., Milosevic K., Zivkovic A., Lavrnja I., Savic D., Bjelobaba I., Janjic M.M. (2024). Alterations in the Hypothalamic-Pituitary-Adrenal Axis as a Response to Experimental Autoimmune Encephalomyelitis in Dark Agouti Rats of Both Sexes. Biomolecules.

[38] Tony M., Plant A.J.Z. (2015). Knobil and Neill’s Physiology of Reproduction.

[39] Wray S. (2010). From nose to brain: Development of gonadotrophin-releasing hormone-1 neurones. J. Neuroendocrinol..

[40] Ebling F.J., Cronin A.S. (2000). The neurobiology of reproductive development. Neuroreport.

[41] Levine J.E., Duffy M.T. (1988). Simultaneous measurement of luteinizing hormone (LH)-releasing hormone, LH, and follicle-stimulating hormone release in intact and short-term castrate rats. Endocrinology.

[42] Park O.K., Ramirez V.D. (1989). Spontaneous changes in LHRH release during the rat estrous cycle, as measured with repetitive push-pull perfusions of the pituitary gland in the same female rats. Neuroendocrinology.

[43] Hall J.E., Strauss J.F., Barbieri R.L. (2019). Chapter 7-Neuroendocrine Control of the Menstrual Cycle. Yen and Jaffe’s Reproductive Endocrinology.

[44] Moenter S.M., Caraty A., Locatelli A., Karsch F.J. (1991). Pattern of gonadotropin-releasing hormone (GnRH) secretion leading up to ovulation in the ewe: Existence of a preovulatory GnRH surge. Endocrinology.

[45] Sarkar D.K., Chiappa S.A., Fink G., Sherwood N.M. (1976). Gonadotropin-releasing hormone surge in pro-oestrous rats. Nature.

[46] Pau K.Y., Berria M., Hess D.L., Spies H.G. (1993). Preovulatory gonadotropin-releasing hormone surge in ovarian-intact rhesus macaques. Endocrinology.

[47] Goodman R.L., Herbison A.E., Lehman M.N., Navarro V.M. (2022). Neuroendocrine control of gonadotropin-releasing hormone: Pulsatile and surge modes of secretion. J. Neuroendocrinol..

[48] Kotani M., Detheux M., Vandenbogaerde A., Communi D., Vanderwinden J.M., Le Poul E., Brézillon S., Tyldesley R., Suarez-Huerta N., Vandeput F. (2001). The metastasis suppressor gene KiSS-1 encodes kisspeptins, the natural ligands of the orphan G protein-coupled receptor GPR54. J. Biol. Chem..

[49] Messager S., Chatzidaki E.E., Ma D., Hendrick A.G., Zahn D., Dixon J., Thresher R.R., Malinge I., Lomet D., Carlton M.B. (2005). Kisspeptin directly stimulates gonadotropin-releasing hormone release via G protein-coupled receptor 54. Proc. Natl. Acad. Sci. USA.

[50] Clarkson J., Herbison A.E. (2006). Postnatal development of kisspeptin neurons in mouse hypothalamus; sexual dimorphism and projections to gonadotropin-releasing hormone neurons. Endocrinology.

[51] Gottsch M.L., Cunningham M.J., Smith J.T., Popa S.M., Acohido B.V., Crowley W.F., Seminara S., Clifton D.K., Steiner R.A. (2004). A role for kisspeptins in the regulation of gonadotropin secretion in the mouse. Endocrinology.

[52] Smith J.T., Dungan H.M., Stoll E.A., Gottsch M.L., Braun R.E., Eacker S.M., Clifton D.K., Steiner R.A. (2005). Differential regulation of KiSS-1 mRNA expression by sex steroids in the brain of the male mouse. Endocrinology.

[53] Clarkson J., d’Anglemont de Tassigny X., Moreno A.S., Colledge W.H., Herbison A.E. (2008). Kisspeptin-GPR54 signaling is essential for preovulatory gonadotropin-releasing hormone neuron activation and the luteinizing hormone surge. J. Neurosci..

[54] Smith J.T., Cunningham M.J., Rissman E.F., Clifton D.K., Steiner R.A. (2005). Regulation of Kiss1 gene expression in the brain of the female mouse. Endocrinology.

[55] Han S.Y., McLennan T., Czieselsky K., Herbison A.E. (2015). Selective optogenetic activation of arcuate kisspeptin neurons generates pulsatile luteinizing hormone secretion. Proc. Natl. Acad. Sci. USA.

[56] Uenoyama Y., Inoue N., Maeda K.I., Tsukamura H. (2018). The roles of kisspeptin in the mechanism underlying reproductive functions in mammals. J. Reprod. Dev..

[57] Nagae M., Uenoyama Y., Okamoto S., Tsuchida H., Ikegami K., Goto T., Majarune S., Nakamura S., Sanbo M., Hirabayashi M. (2021). Direct evidence that KNDy neurons maintain gonadotropin pulses and folliculogenesis as the GnRH pulse generator. Proc. Natl. Acad. Sci. USA.

[58] Marques P., De Sousa Lages A., Skorupskaite K., Rozario K.S., Anderson R.A., George J.T., Feingold K.R., Adler R.A., Ahmed S.F., Anawalt B., Blackman M.R., Chrousos G., Corpas E., de Herder W.W., Dhatariya K., Dungan K. (2000). Physiology of GnRH and Gonadotrophin Secretion. Endotext.

[59] McArdle C.A., Roberson M.S., Plant T.M., Zeleznik A.J. (2015). Chapter 10-Gonadotropes and Gonadotropin-Releasing Hormone Signaling. Knobil and Neill’s Physiology of Reproduction.

[60] Narayan P., Ulloa-Aguirre A., Dias J.A., Strauss J.F., Barbieri R.L. (2019). Chapter 2-Gonadotropin Hormones and Their Receptors. Yen and Jaffe’s Reproductive Endocrinology.

[61] Bliss S.P., Navratil A.M., Xie J., Roberson M.S. (2010). GnRH signaling, the gonadotrope and endocrine control of fertility. Front. Neuroendocrinol..

[62] Kaiser U.B., Jakubowiak A., Steinberger A., Chin W.W. (1997). Differential effects of gonadotropin-releasing hormone (GnRH) pulse frequency on gonadotropin subunit and GnRH receptor messenger ribonucleic acid levels in vitro. Endocrinology.

[63] Kaprara A., Huhtaniemi I.T. (2018). The hypothalamus-pituitary-gonad axis: Tales of mice and men. Metabolism.

[64] Anderson L. (1996). Intracellular mechanisms triggering gonadotrophin secretion. Rev. Reprod..

[65] Rone M.B., Fan J., Papadopoulos V. (2009). Cholesterol transport in steroid biosynthesis: Role of protein-protein interactions and implications in disease states. Biochim. Biophys. Acta.

[66] Payne A.H., Hales D.B. (2004). Overview of steroidogenic enzymes in the pathway from cholesterol to active steroid hormones. Endocr. Rev..

[67] Zirkin B.R., Papadopoulos V. (2018). Leydig cells: Formation, function, and regulation. Biol. Reprod..

[68] Clark B.J., Wells J., King S.R., Stocco D.M. (1994). The purification, cloning, and expression of a novel luteinizing hormone-induced mitochondrial protein in MA-10 mouse Leydig tumor cells. Characterization of the steroidogenic acute regulatory protein (StAR). J. Biol. Chem..

[69] Plant T.M. (2015). 60 YEARS OF NEUROENDOCRINOLOGY: The hypothalamo-pituitary-gonadal axis. J. Endocrinol..

[70] Plant T.M. (2012). A comparison of the neuroendocrine mechanisms underlying the initiation of the preovulatory LH surge in the human, Old World monkey and rodent. Front. Neuroendocrinol..

[71] Koebele S.V., Bimonte-Nelson H.A. (2016). Modeling menopause: The utility of rodents in translational behavioral endocrinology research. Maturitas.

[72] Walton C., King R., Rechtman L., Kaye W., Leray E., Marrie R.A., Robertson N., La Rocca N., Uitdehaag B., van der Mei I. (2020). Rising prevalence of multiple sclerosis worldwide: Insights from the Atlas of MS, third edition. Mult. Scler..

[73] Libert C., Dejager L., Pinheiro I. (2010). The X chromosome in immune functions: When a chromosome makes the difference. Nat. Rev. Immunol..

[74] Gilli F., DiSano K.D., Pachner A.R. (2020). SeXX Matters in Multiple Sclerosis. Front. Neurol..

[75] Pozzilli C., Tomassini V., Marinelli F., Paolillo A., Gasperini C., Bastianello S. (2003). ‘Gender gap’ in multiple sclerosis: Magnetic resonance imaging evidence. Eur. J. Neurol..

[76] Bove R., Chitnis T. (2013). Sexual disparities in the incidence and course of MS. Clin. Immunol..

[77] Chitnis T. (2018). The role of testosterone in MS risk and course. Mult. Scler..

[78] Magyari M., Koch-Henriksen N. (2022). Quantitative effect of sex on disease activity and disability accumulation in multiple sclerosis. J. Neurol. Neurosurg. Psychiatry.

[79] Ysrraelit M.C., Correale J. (2019). Impact of sex hormones on immune function and multiple sclerosis development. Immunology.

[80] Collongues N., Patte-Mensah C., De Seze J., Mensah-Nyagan A.G., Derfuss T. (2018). Testosterone and estrogen in multiple sclerosis: From pathophysiology to therapeutics. Expert. Rev. Neurother..

[81] Confavreux C., Vukusic S., Adeleine P. (2003). Early clinical predictors and progression of irreversible disability in multiple sclerosis: An amnesic process. Brain.

[82] Malik M.T., Healy B.C., Benson L.A., Kivisakk P., Musallam A., Weiner H.L., Chitnis T. (2014). Factors associated with recovery from acute optic neuritis in patients with multiple sclerosis. Neurology.

[83] Papenfuss T.L., Rogers C.J., Gienapp I., Yurrita M., McClain M., Damico N., Valo J., Song F., Whitacre C.C. (2004). Sex differences in experimental autoimmune encephalomyelitis in multiple murine strains. J. Neuroimmunol..

[84] Bebo B.F., Schuster J.C., Vandenbark A.A., Offner H. (1998). Gender differences in experimental autoimmune encephalomyelitis develop during the induction of the immune response to encephalitogenic peptides. J. Neurosci. Res..

[85] Bebo B.F., Schuster J.C., Vandenbark A.A., Offner H. (1999). Androgens alter the cytokine profile and reduce encephalitogenicity of myelin-reactive T cells. J. Immunol..

[86] Massilamany C., Thulasingam S., Steffen D., Reddy J. (2011). Gender differences in CNS autoimmunity induced by mimicry epitope for PLP 139-151 in SJL mice. J. Neuroimmunol..

[87] Nacka-Aleksić M., Djikić J., Pilipović I., Stojić-Vukanić Z., Kosec D., Bufan B., Arsenović-Ranin N., Dimitrijević M., Leposavić G. (2015). Male rats develop more severe experimental autoimmune encephalomyelitis than female rats: Sexual dimorphism and diergism at the spinal cord level. Brain Behav. Immun..

[88] Stojić-Vukanić Z., Kotur-Stevuljević J., Nacka-Aleksić M., Kosec D., Vujnović I., Pilipović I., Dimitrijević M., Leposavić G. (2018). Sex Bias in Pathogenesis of Autoimmune Neuroinflammation: Relevance for Dimethyl Fumarate Immunomodulatory/Anti-oxidant Action. Mol. Neurobiol..

[89] Fillmore P.D., Blankenhorn E.P., Zachary J.F., Teuscher C. (2004). Adult gonadal hormones selectively regulate sexually dimorphic quantitative traits observed in experimental allergic encephalomyelitis. Am. J. Pathol..

[90] Wiedrick J., Meza-Romero R., Gerstner G., Seifert H., Chaudhary P., Headrick A., Kent G., Maestas A., Offner H., Vandenbark A.A. (2021). Sex differences in EAE reveal common and distinct cellular and molecular components. Cell Immunol..

[91] Giatti S., Rigolio R., Diviccaro S., Falvo E., Caruso D., Garcia-Segura L.M., Cavaletti G., Melcangi R.C. (2020). Sex dimorphism in an animal model of multiple sclerosis: Focus on pregnenolone synthesis. J. Steroid Biochem. Mol. Biol..

[92] Bjelobaba I., Begovic-Kupresanin V., Pekovic S., Lavrnja I. (2018). Animal models of multiple sclerosis: Focus on experimental autoimmune encephalomyelitis. J. Neurosci. Res..

[93] Keith A.B. (1978). Sex difference in Lewis rats in the incidence of recurrent experimental allergic encephalomyelitis. Nature.

[94] Gupta S., Arnab S., Stehle E., Nguyen K.L. (2026). Advancing EAE Modeling: Establishment of a Non-Pertussis Immunization Protocol for Multiple Sclerosis. Bio-Protocol.

[95] Murphy K.L., Fischer R., Swanson K.A., Bhatt I.J., Oakley L., Smeyne R., Bracchi-Ricard V., Bethea J.R. (2020). Synaptic alterations and immune response are sexually dimorphic in a non-pertussis toxin model of experimental autoimmune encephalomyelitis. Exp. Neurol..

[96] Spence R.D., Voskuhl R.R. (2012). Neuroprotective effects of estrogens and androgens in CNS inflammation and neurodegeneration. Front. Neuroendocrinol..

[97] Ahn J.J., O’Mahony J., Moshkova M., Hanwell H.E., Singh H., Zhang M.A., Marrie R.A., Bar-Or A., Sadovnick D.A., Dunn S.E. (2015). Puberty in females enhances the risk of an outcome of multiple sclerosis in children and the development of central nervous system autoimmunity in mice. Mult. Scler..

[98] Bove R. (2016). Women’s Issues in Multiple Sclerosis. Semin. Neurol..

[99] Ramagopalan S.V., Valdar W., Criscuoli M., DeLuca G.C., Dyment D.A., Orton S.M., Yee I.M., Ebers G.C., Sadovnick A.D. (2009). Age of puberty and the risk of multiple sclerosis: A population based study. Eur. J. Neurol..

[100] Sloka J.S., Pryse-Phillips W.E., Stefanelli M. (2006). The relation between menarche and the age of first symptoms in a multiple sclerosis cohort. Mult. Scler..

[101] McCombe P.A. (2018). The Short and Long-Term Effects of Pregnancy on Multiple Sclerosis and Experimental Autoimmune Encephalomyelitis. J. Clin. Med..

[102] Langer-Gould A., Smith J.B., Albers K.B., Xiang A.H., Wu J., Kerezsi E.H., McClearnen K., Gonzales E.G., Leimpeter A.D., Van Den Eeden S.K. (2020). Pregnancy-related relapses and breastfeeding in a contemporary multiple sclerosis cohort. Neurology.

[103] Confavreux C., Hutchinson M., Hours M.M., Cortinovis-Tourniaire P., Moreau T. (1998). Rate of pregnancy-related relapse in multiple sclerosis. Pregnancy in Multiple Sclerosis Group. N. Engl. J. Med..

[104] Verdru P., Theys P., D’Hooghe M.B., Carton H. (1994). Pregnancy and multiple sclerosis: The influence on long term disability. Clin. Neurol. Neurosurg..

[105] Masera S., Cavalla P., Prosperini L., Mattioda A., Mancinelli C.R., Superti G., Chiavazza C., Vercellino M., Pinessi L., Pozzilli C. (2015). Parity is associated with a longer time to reach irreversible disability milestones in women with multiple sclerosis. Mult. Scler..

[106] D’Hooghe M.B., Haentjens P., Nagels G., D’Hooghe T., De Keyser J. (2012). Menarche, oral contraceptives, pregnancy and progression of disability in relapsing onset and progressive onset multiple sclerosis. J. Neurol..

[107] Langer-Gould A., Smith J.B., Hellwig K., Gonzales E., Haraszti S., Koebnick C., Xiang A. (2017). Breastfeeding, ovulatory years, and risk of multiple sclerosis. Neurology.

[108] Bove R., Okai A., Houtchens M., Elias-Hamp B., Lugaresi A., Hellwig K., Kubala Havrdová E. (2021). Effects of Menopause in Women with Multiple Sclerosis: An Evidence-Based Review. Front. Neurol..

[109] Graves J.S., Henry R.G., Cree B.A.C., Lambert-Messerlian G., Greenblatt R.M., Waubant E., Cedars M.I., Zhu A., Bacchetti P., Hauser S.L. (2018). Ovarian aging is associated with gray matter volume and disability in women with MS. Neurology.

[110] Bove R., Malik M.T., Diaz-Cruz C., Chua A., Saraceno T.J., Bargiela D., Greeke E., Glanz B.I., Healy B.C., Chitnis T. (2015). The 2D:4D ratio, a proxy for prenatal androgen levels, differs in men with and without MS. Neurology.

[111] Ysrraelit M.C., Correale J. (2021). Impact of Andropause on Multiple Sclerosis. Front. Neurol..

[112] Pakpoor J., Wotton C.J., Schmierer K., Giovannoni G., Goldacre M.J. (2016). Gender identity disorders and multiple sclerosis risk: A national record-linkage study. Mult. Scler..

[113] Hsu S., Bove R. (2024). Hormonal Therapies in Multiple Sclerosis: A Review of Clinical Data. Curr. Neurol. Neurosci. Rep..

[114] Bebo B.F., Zelinka-Vincent E., Adamus G., Amundson D., Vandenbark A.A., Offner H. (1998). Gonadal hormones influence the immune response to PLP 139-151 and the clinical course of relapsing experimental autoimmune encephalomyelitis. J. Neuroimmunol..

[115] Palaszynski K.M., Loo K.K., Ashouri J.F., Liu H.B., Voskuhl R.R. (2004). Androgens are protective in experimental autoimmune encephalomyelitis: Implications for multiple sclerosis. J. Neuroimmunol..

[116] Trooster W.J., Teelken A.W., Gerrits P.O., Lijnema T.H., Loof J.G., Minderhoud J.M., Nieuwenhuis P. (1996). The effect of gonadectomy on the clinical course of chronic experimental allergic encephalomyelitis. Clin. Neurol. Neurosurg..

[117] Macció D.R., Ditamo Y., Degano A.L., Roth G.A. (2004). Interaction between gonadal steroids and neuroimmune system in acute experimental autoimmune encephalomyelitis (EAE) in Wistar rats. Autoimmunity.

[118] Langer-Gould A., Garren H., Slansky A., Ruiz P.J., Steinman L. (2002). Late pregnancy suppresses relapses in experimental autoimmune encephalomyelitis: Evidence for a suppressive pregnancy-related serum factor. J. Immunol..

[119] McClain M.A., Gatson N.N., Powell N.D., Papenfuss T.L., Gienapp I.E., Song F., Shawler T.M., Kithcart A., Whitacre C.C. (2007). Pregnancy suppresses experimental autoimmune encephalomyelitis through immunoregulatory cytokine production. J. Immunol..

[120] Harness J., McCombe P.A. (2001). The effects of pregnancy on myelin basic protein-induced experimental autoimmune encephalomyelitis in Lewis rats: Suppression of clinical disease, modulation of cytokine expression in the spinal cord inflammatory infiltrate and suppression of lymphocyte proliferation by pregnancy sera. Am. J. Reprod. Immunol..

[121] Keith A.B. (1978). Effect of pregnancy on experimental allergic encephalomyelitis in guinea pigs and rats. J. Neurol. Sci..

[122] Mertin L.A., Rumjanek V.M. (1985). Pregnancy and the susceptibility of Lewis rats to experimental allergic encephalomyelitis. J. Neurol. Sci..

[123] Gatson N.N., Williams J.L., Powell N.D., McClain M.A., Hennon T.R., Robbins P.D., Whitacre C.C. (2011). Induction of pregnancy during established EAE halts progression of CNS autoimmune injury via pregnancy-specific serum factors. J. Neuroimmunol..

[124] Engler J.B., Kursawe N., Solano M.E., Patas K., Wehrmann S., Heckmann N., Lühder F., Reichardt H.M., Arck P.C., Gold S.M. (2017). Glucocorticoid receptor in T cells mediates protection from autoimmunity in pregnancy. Proc. Natl. Acad. Sci. USA.

[125] Jansson L., Olsson T., Holmdahl R. (1994). Estrogen induces a potent suppression of experimental autoimmune encephalomyelitis and collagen-induced arthritis in mice. J. Neuroimmunol..

[126] Nashold F.E., Spach K.M., Spanier J.A., Hayes C.E. (2009). Estrogen controls vitamin D3-mediated resistance to experimental autoimmune encephalomyelitis by controlling vitamin D3 metabolism and receptor expression. J. Immunol..

[127] Haghmorad D., Salehipour Z., Nosratabadi R., Rastin M., Kokhaei P., Mahmoudi M.B., Amini A.A., Mahmoudi M. (2016). Medium-dose estrogen ameliorates experimental autoimmune encephalomyelitis in ovariectomized mice. J. Immunotoxicol..

[128] Lélu K., Delpy L., Robert V., Foulon E., Laffont S., Pelletier L., Engelhardt B., Guéry J.C. (2010). Endogenous estrogens, through estrogen receptor α, constrain autoimmune inflammation in female mice by limiting CD4+ T-cell homing into the CNS. Eur. J. Immunol..

[129] Smith-Bouvier D.L., Divekar A.A., Sasidhar M., Du S., Tiwari-Woodruff S.K., King J.K., Arnold A.P., Singh R.R., Voskuhl R.R. (2008). A role for sex chromosome complement in the female bias in autoimmune disease. J. Exp. Med..

[130] Du S., Itoh N., Askarinam S., Hill H., Arnold A.P., Voskuhl R.R. (2014). XY sex chromosome complement, compared with XX, in the CNS confers greater neurodegeneration during experimental autoimmune encephalomyelitis. Proc. Natl. Acad. Sci. USA.

[131] Golden L.C., Itoh Y., Itoh N., Iyengar S., Coit P., Salama Y., Arnold A.P., Sawalha A.H., Voskuhl R.R. (2019). Parent-of-origin differences in DNA methylation of X chromosome genes in T lymphocytes. Proc. Natl. Acad. Sci. USA.

[132] Gupta S., Arnab S., Nguyen K.L., Reed M., Fathi P., Tammen K., Turner E., Jones E., Fischer R., Mendelowitz D. (2025). Sex-chromosome complement and Activin-A shape the therapeutic potential of TNFR2 activation in a model of MS and CNP. Proc. Natl. Acad. Sci. USA.

[133] Zorgdrager A., De Keyser J. (1997). Menstrually related worsening of symptoms in multiple sclerosis. J. Neurol. Sci..

[134] Zorgdrager A., De Keyser J. (2002). The premenstrual period and exacerbations in multiple sclerosis. Eur. Neurol..

[135] Tomassini V., Onesti E., Mainero C., Giugni E., Paolillo A., Salvetti M., Nicoletti F., Pozzilli C. (2005). Sex hormones modulate brain damage in multiple sclerosis: MRI evidence. J. Neurol. Neurosurg. Psychiatry.

[136] Kempe P., Eklund D., Hallin A., Hammar M., Olsson T., Brynhildsen J., Ernerudh J. (2018). Immune profile in relation to sex steroid cyclicity in healthy women and women with multiple sclerosis. J. Reprod. Immunol..

[137] Foroughipour A., Norbakhsh V., Najafabadi S.H., Meamar R. (2012). Evaluating sex hormone levels in reproductive age women with multiple sclerosis and their relationship with disease severity. J. Res. Med. Sci..

[138] Guven Yorgun Y., Ozakbas S. (2019). Effect of hormonal changes on the neurological status in the menstrual cycle of patient with multiple sclerosis. Clin. Neurol. Neurosurg..

[139] Sepúlveda M., Ros C., Martínez-Lapiscina E.H., Solà-Valls N., Hervàs M., Llufriu S., La Puma D., Casals E., Blanco Y., Villoslada P. (2016). Pituitary-ovary axis and ovarian reserve in fertile women with multiple sclerosis: A pilot study. Mult. Scler..

[140] Caruso D., Melis M., Fenu G., Giatti S., Romano S., Grimoldi M., Crippa D., Marrosu M.G., Cavaletti G., Melcangi R.C. (2014). Neuroactive steroid levels in plasma and cerebrospinal fluid of male multiple sclerosis patients. J. Neurochem..

[141] Bansil S., Lee H.J., Jindal S., Holtz C.R., Cook S.D. (1999). Correlation between sex hormones and magnetic resonance imaging lesions in multiple sclerosis. Acta Neurol. Scand..

[142] Pozzilli C., Falaschi P., Mainero C., Martocchia A., D’Urso R., Proietti A., Frontoni M., Bastianello S., Filippi M. (1999). MRI in multiple sclerosis during the menstrual cycle: Relationship with sex hormone patterns. Neurology.

[143] Wei T., Lightman S.L. (1997). The neuroendocrine axis in patients with multiple sclerosis. Brain.

[144] D’Amico E., Zanghì A., Burgio G., Chisari C.G., Condorelli R.A., La Vignera S., Calogero A.E., Patti F. (2020). Gonadal Steroids and Sperm Quality in a Cohort of Relapsing Remitting Multiple Sclerosis: A Case-Control Study. Front. Neurol..

[145] Safarinejad M.R. (2008). Evaluation of endocrine profile, hypothalamic-pituitary-testis axis and semen quality in multiple sclerosis. J. Neuroendocrinol..

[146] Bove R., Musallam A., Healy B.C., Raghavan K., Glanz B.I., Bakshi R., Weiner H., De Jager P.L., Miller K.K., Chitnis T. (2014). Low testosterone is associated with disability in men with multiple sclerosis. Mult. Scler..

[147] Grinsted L., Heltberg A., Hagen C., Djursing H. (1989). Serum sex hormone and gonadotropin concentrations in premenopausal women with multiple sclerosis. J. Intern. Med..

[148] Rahn E.J., Iannitti T., Donahue R.R., Taylor B.K. (2014). Sex differences in a mouse model of multiple sclerosis: Neuropathic pain behavior in females but not males and protection from neurological deficits during proestrus. Biol. Sex. Differ..

[149] Jaini R., Altuntas C.Z., Loya M.G., Tuohy V.K. (2015). Disruption of estrous cycle homeostasis in mice with experimental autoimmune encephalomyelitis. J. Neuroimmunol..

[150] Taylor L.C., Gilmore W., Ting J.P., Matsushima G.K. (2010). Cuprizone induces similar demyelination in male and female C57BL/6 mice and results in disruption of the estrous cycle. J. Neurosci. Res..

[151] Giatti S., D’Intino G., Maschi O., Pesaresi M., Garcia-Segura L.M., Calza L., Caruso D., Melcangi R.C. (2010). Acute experimental autoimmune encephalomyelitis induces sex dimorphic changes in neuroactive steroid levels. Neurochem. Int..

[152] Caruso D., D’Intino G., Giatti S., Maschi O., Pesaresi M., Calabrese D., Garcia-Segura L.M., Calza L., Melcangi R.C. (2010). Sex-dimorphic changes in neuroactive steroid levels after chronic experimental autoimmune encephalomyelitis. J. Neurochem..

[153] Handa R.J., Sheng J.A., Castellanos E.A., Templeton H.N., McGivern R.F. (2022). Sex Differences in Acute Neuroendocrine Responses to Stressors in Rodents and Humans. Cold Spring Harb. Perspect. Biol..

[154] de Kloet E.R. (2024). Coping with the multifaceted and multifunctional role of cortisol in the brain. Neurosci. Appl..

[155] Moriguchi K., Miyamoto K., Fukumoto Y., Kusunoki S. (2021). Change in light-dark cycle affects experimental autoimmune encephalomyelitis. J. Neuroimmunol..

[156] Jiang Y., Liu C., Zhong Y. (2025). Temporal Orchestration of Immunity: How Circadian Clocks Coordinate Immune Responses. Front. Biosci..

[157] Pivovarova-Ramich O., Zimmermann H.G., Paul F. (2023). Multiple sclerosis and circadian rhythms: Can diet act as a treatment?. Acta Physiol..

[158] Bjelobaba I., Savic D., Lavrnja I. (2017). Multiple Sclerosis and Neuroinflammation: The Overview of Current and Prospective Therapies. Curr. Pharm. Des..

[159] Bebo B.F., Fyfe-Johnson A., Adlard K., Beam A.G., Vandenbark A.A., Offner H. (2001). Low-dose estrogen therapy ameliorates experimental autoimmune encephalomyelitis in two different inbred mouse strains. J. Immunol..

[160] Subramanian S., Matejuk A., Zamora A., Vandenbark A.A., Offner H. (2003). Oral feeding with ethinyl estradiol suppresses and treats experimental autoimmune encephalomyelitis in SJL mice and inhibits the recruitment of inflammatory cells into the central nervous system. J. Immunol..

[161] Ito A., Bebo B.F., Matejuk A., Zamora A., Silverman M., Fyfe-Johnson A., Offner H. (2001). Estrogen treatment down-regulates TNF-alpha production and reduces the severity of experimental autoimmune encephalomyelitis in cytokine knockout mice. J. Immunol..

[162] Matejuk A., Adlard K., Zamora A., Silverman M., Vandenbark A.A., Offner H. (2001). 17 beta-estradiol inhibits cytokine, chemokine, and chemokine receptor mRNA expression in the central nervous system of female mice with experimental autoimmune encephalomyelitis. J. Neurosci. Res..

[163] Polanczyk M., Zamora A., Subramanian S., Matejuk A., Hess D.L., Blankenhorn E.P., Teuscher C., Vandenbark A.A., Offner H. (2003). The protective effect of 17beta-estradiol on experimental autoimmune encephalomyelitis is mediated through estrogen receptor-alpha. Am. J. Pathol..

[164] Tiwari-Woodruff S., Morales L.B., Lee R., Voskuhl R.R. (2007). Differential neuroprotective and antiinflammatory effects of estrogen receptor (ER)alpha and ERbeta ligand treatment. Proc. Natl. Acad. Sci. USA.

[165] De Giglio L., Marinelli F., Barletta V.T., Pagano V.A., De Angelis F., Fanelli F., Petsas N., Pantano P., Tomassini V., Pozzilli C. (2017). Effect on Cognition of Estroprogestins Combined with Interferon Beta in Multiple Sclerosis: Analysis of Secondary Outcomes from a Randomised Controlled Trial. CNS Drugs.

[166] Pozzilli C., De Giglio L., Barletta V.T., Marinelli F., Angelis F.D., Gallo V., Pagano V.A., Marini S., Piattella M.C., Tomassini V. (2015). Oral contraceptives combined with interferon β in multiple sclerosis. Neurol. Neuroimmunol. Neuroinflamm..

[167] Palaszynski K.M., Liu H., Loo K.K., Voskuhl R.R. (2004). Estriol treatment ameliorates disease in males with experimental autoimmune encephalomyelitis: Implications for multiple sclerosis. J. Neuroimmunol..

[168] Kim S., Liva S.M., Dalal M.A., Verity M.A., Voskuhl R.R. (1999). Estriol ameliorates autoimmune demyelinating disease: Implications for multiple sclerosis. Neurology.

[169] Sicotte N.L., Liva S.M., Klutch R., Pfeiffer P., Bouvier S., Odesa S., Wu T.C., Voskuhl R.R. (2002). Treatment of multiple sclerosis with the pregnancy hormone estriol. Ann. Neurol..

[170] Soldan S.S., Alvarez Retuerto A.I., Sicotte N.L., Voskuhl R.R. (2003). Immune modulation in multiple sclerosis patients treated with the pregnancy hormone estriol. J. Immunol..

[171] Voskuhl R.R., Wang H., Wu T.C., Sicotte N.L., Nakamura K., Kurth F., Itoh N., Bardens J., Bernard J.T., Corboy J.R. (2016). Estriol combined with glatiramer acetate for women with relapsing-remitting multiple sclerosis: A randomised, placebo-controlled, phase 2 trial. Lancet Neurol..

[172] MacKenzie-Graham A., Brook J., Kurth F., Itoh Y., Meyer C., Montag M.J., Wang H.J., Elashoff R., Voskuhl R.R. (2018). Estriol-mediated neuroprotection in multiple sclerosis localized by voxel-based morphometry. Brain Behav..

[173] Giatti S., Caruso D., Boraso M., Abbiati F., Ballarini E., Calabrese D., Pesaresi M., Rigolio R., Santos-Galindo M., Viviani B. (2012). Neuroprotective effects of progesterone in chronic experimental autoimmune encephalomyelitis. J. Neuroendocrinol..

[174] Yates M.A., Li Y., Chlebeck P., Proctor T., Vandenbark A.A., Offner H. (2010). Progesterone treatment reduces disease severity and increases IL-10 in experimental autoimmune encephalomyelitis. J. Neuroimmunol..

[175] Garay L., Gonzalez Deniselle M.C., Meyer M., Costa J.J., Lima A., Roig P., De nicola A.F. (2009). Protective effects of progesterone administration on axonal pathology in mice with experimental autoimmune encephalomyelitis. Brain Res..

[176] El-Etr M., Rame M., Boucher C., Ghoumari A.M., Kumar N., Liere P., Pianos A., Schumacher M., Sitruk-Ware R. (2015). Progesterone and nestorone promote myelin regeneration in chronic demyelinating lesions of corpus callosum and cerebral cortex. Glia.

[177] Ibanez C., Shields S.A., El-Etr M., Baulieu E.E., Schumacher M., Franklin R.J. (2004). Systemic progesterone administration results in a partial reversal of the age-associated decline in CNS remyelination following toxin-induced demyelination in male rats. Neuropathol. Appl. Neurobiol..

[178] Garay L., Gonzalez Deniselle M.C., Lima A., Roig P., De Nicola A.F. (2007). Effects of progesterone in the spinal cord of a mouse model of multiple sclerosis. J. Steroid Biochem. Mol. Biol..

[179] Garay L., Gonzalez Deniselle M.C., Gierman L., Lima A., Roig P., De Nicola A.F. (2010). Pharmacotherapy with 17β-estradiol and progesterone prevents development of mouse experimental autoimmune encephalomyelitis. Horm. Mol. Biol. Clin. Investig..

[180] Hoffman G.E., Le W.W., Murphy A.Z., Koski C.L. (2001). Divergent effects of ovarian steroids on neuronal survival during experimental allergic encephalitis in Lewis rats. Exp. Neurol..

[181] Vukusic S., Ionescu I., Cornu C., Bossard N., Durand-Dubief F., Cotton F. (2021). Oral nomegestrol acetate and transdermal 17-beta-estradiol for preventing post-partum relapses in multiple sclerosis: The POPARTMUS study. Mult. Scler. J..

[182] Gubbels Bupp M.R., Jorgensen T.N. (2018). Androgen-Induced Immunosuppression. Front. Immunol..

[183] Dalal M., Kim S., Voskuhl R.R. (1997). Testosterone therapy ameliorates experimental autoimmune encephalomyelitis and induces a T helper 2 bias in the autoantigen-specific T lymphocyte response. J. Immunol..

[184] Liva S.M., Voskuhl R.R. (2001). Testosterone acts directly on CD4+ T lymphocytes to increase IL-10 production. J. Immunol..

[185] Macció D.R., Calfa G., Roth G.A. (2005). Oral testosterone in male rats and the development of experimental autoimmune encephalomyelitis. Neuroimmunomodulation.

[186] Giatti S., Rigolio R., Romano S., Mitro N., Viviani B., Cavaletti G., Caruso D., Garcia-Segura L.M., Melcangi R.C. (2015). Dihydrotestosterone as a Protective Agent in Chronic Experimental Autoimmune Encephalomyelitis. Neuroendocrinology.

[187] Ziehn M.O., Avedisian A.A., Dervin S.M., Umeda E.A., O’Dell T.J., Voskuhl R.R. (2012). Therapeutic testosterone administration preserves excitatory synaptic transmission in the hippocampus during autoimmune demyelinating disease. J. Neurosci..

[188] Patel R., Moore S., Crawford D.K., Hannsun G., Sasidhar M.V., Tan K., Molaie D., Tiwari-Woodruff S.K. (2013). Attenuation of corpus callosum axon myelination and remyelination in the absence of circulating sex hormones. Brain Pathol..

[189] Bielecki B., Mattern C., Ghoumari A.M., Javaid S., Smietanka K., Abi Ghanem C., Mhaouty-Kodja S., Ghandour M.S., Baulieu E.E., Franklin R.J. (2016). Unexpected central role of the androgen receptor in the spontaneous regeneration of myelin. Proc. Natl. Acad. Sci. USA.

[190] Hussain R., Ghoumari A.M., Bielecki B., Steibel J., Boehm N., Liere P., Macklin W.B., Kumar N., Habert R., Mhaouty-Kodja S. (2013). The neural androgen receptor: A therapeutic target for myelin repair in chronic demyelination. Brain.

[191] Sicotte N.L., Giesser B.S., Tandon V., Klutch R., Steiner B., Drain A.E., Shattuck D.W., Hull L., Wang H.J., Elashoff R.M. (2007). Testosterone treatment in multiple sclerosis: A pilot study. Arch. Neurol..

[192] Kurth F., Luders E., Sicotte N.L., Gaser C., Giesser B.S., Swerdloff R.S., Montag M.J., Voskuhl R.R., Mackenzie-Graham A. (2014). Neuroprotective effects of testosterone treatment in men with multiple sclerosis. Neuroimage Clin..

[193] Gold S.M., Chalifoux S., Giesser B.S., Voskuhl R.R. (2008). Immune modulation and increased neurotrophic factor production in multiple sclerosis patients treated with testosterone. J. Neuroinflammation.

[194] Metzger-Peter K., Kremer L.D., Edan G., Loureiro De Sousa P., Lamy J., Bagnard D., Mensah-Nyagan A.G., Tricard T., Mathey G., Debouverie M. (2020). The TOTEM RRMS (Testosterone Treatment on neuroprotection and Myelin Repair in Relapsing Remitting Multiple Sclerosis) trial: Study protocol for a randomized, double-blind, placebo-controlled trial. Trials.

[195] Zeydan B., Neyal N., Nathoo N., Rangachari M., Atkinson E.J., Son J., Conway B.L., Tobin W.O., Keegan B.M., Weinshenker B.G. (2025). Effects of androgen modifying therapies on disease activity in older men with multiple sclerosis. J. Neuroimmunol..

[196] Traiffort E., Kassoussi A., Zahaf A. (2025). Revisiting the role of sexual hormones in the demyelinated central nervous system. Front. Neuroendocrinol..

